# Interindividual methylomic variation across blood, cortex, and cerebellum: implications for epigenetic studies of neurological and neuropsychiatric phenotypes

**DOI:** 10.1080/15592294.2015.1100786

**Published:** 2015-10-12

**Authors:** Eilis Hannon, Katie Lunnon, Leonard Schalkwyk, Jonathan Mill

**Affiliations:** 1 University of Exeter Medical School; University of Exeter; Devon, UK; 2 School of Biological Sciences; University of Essex; Wivenhoe Park, Colchester, UK; 3 Institute of Psychiatry; Psychology & Neuroscience; King's College London; De Crespigny Park, London, UK

**Keywords:** brain, blood, cortex, cerebellum, DNA methylation, epigenetic epidemiology, Illumina 450K array

## Abstract

Given the tissue-specific nature of epigenetic processes, the assessment of disease-relevant tissue is an important consideration for epigenome-wide association studies (EWAS). Little is known about whether easily accessible tissues, such as whole blood, can be used to address questions about interindividual epigenomic variation in inaccessible tissues, such as the brain. We quantified DNA methylation in matched DNA samples isolated from whole blood and 4 brain regions (prefrontal cortex, entorhinal cortex, superior temporal gyrus, and cerebellum) from 122 individuals. We explored co-variation between tissues and the extent to which methylomic variation in blood is predictive of interindividual variation identified in the brain. For the majority of DNA methylation sites, interindividual variation in whole blood is not a strong predictor of interindividual variation in the brain, although the relationship with cortical regions is stronger than with the cerebellum. Variation at a subset of probes is strongly correlated across tissues, even in instances when the actual level of DNA methylation is significantly different between them. A substantial proportion of this co-variation, however, is likely to result from genetic influences. Our data suggest that for the majority of the genome, a blood-based EWAS for disorders where brain is presumed to be the primary tissue of interest will give limited information relating to underlying pathological processes. These results do not, however, discount the utility of using a blood-based EWAS to identify biomarkers of disease phenotypes manifest in the brain. We have generated a searchable database for the interpretation of data from blood-based EWAS analyses (http://epigenetics.essex.ac.uk/bloodbrain/).

## Introduction

There is increasing interest in the role of epigenetic processes in health and disease, with the primary focus of most epigenetic epidemiological studies to date being DNA methylation.^1^ Platforms such as the Illumina Infinium HumanMethylation450 BeadChip (450K) have enabled the economical, high-throughput profiling of methylomic variation across large numbers of samples and epigenome-wide association studies (EWAS), which aim to identify DNA methylation differences associated with environmental exposure and disease, are now underway for many types of pathology, including cancer,^2-4^ autoimmune disorders,^5,6^ psychiatric phenotypes,^7^ neurodevelopmental disorders,^8,9^ and dementia.^10,11^ Despite the recent successes in identifying disease-associated epigenetic variation, the design, analysis, and interpretation of EWAS requires careful attention; there are a number of critical issues that need to be considered in epigenetic epidemiology that preclude a simple re-analysis of DNA samples collected for genome-wide association studies (GWAS).^9,12-14^

Of particular importance is the fact that, unlike germline genetic variation, epigenetic signatures are tissue-specific; therefore, the selection of tissue type for epigenetic epidemiology is potentially critical. The ENCODE and the NIH Epigenomics Roadmap projects,^15-17^ for example, have recently characterized the distinct epigenetic profiles defining human cell-types, highlighting how these reflect the developmental relationships between them. It is clear that intraindividual epigenetic differences (i.e., between tissues within a single person) greatly outweigh interindividual differences within a specific tissue type.^18-23^ Although many clinical and epidemiological studies are examining epigenetic variation in easily accessible cells obtained from tissues, such as whole blood, the extent to which these can be used to address questions about interindividual epigenomic variation in inaccessible tissues, such as the brain, has not yet been systematically explored. Addressing this issue will be critical given the paucity of high-quality brain tissue from clinically well-phenotyped patients and controls, especially if EWAS analyses require sample sizes approaching those necessary to identify genetic associations with complex disease phenotypes. Because the brain and blood originate from different developmental cell lineages and are epigenetically distinct,^23^ it is clearly inappropriate to use blood as a proxy measure for actual brain DNA methylation profiles. Despite this, epidemiological studies using accessible peripheral tissues may still be informative in an epidemiological context if interindividual *variation* is correlated across tissues.

In this study we quantified DNA methylation using the 450K array in a collection of matched DNA samples isolated from pre-mortem whole blood and 4 post-mortem brain regions [prefrontal cortex (PFC), entorhinal cortex (EC), superior temporal gyrus (STG), and cerebellum (CER)] dissected at autopsy from 122 individuals. We describe patterns of co-variation across tissues and identify sites where estimates of DNA methylation in whole blood are predictive of interindividual variation in DNA methylation across the 4 brain regions. Our data are available in an online searchable database (http://epigenetics.essex.ac.uk/bloodbrain/) to enable the research community to explore the relationship between whole blood and brain DNA methylation patterns at specific locations across the genome.

## Results and Discussion

### Cortex, cerebellum, and blood are defined by very distinct profiles of DNA methylation

We used the Illumina 450K array to quantify DNA methylation in 4 dissected brain regions (PFC: n = 114, EC: n = 108, STG: n = 117 and CER: n = 112) and matched pre-mortem whole blood samples (n = 80) from an overlapping set of 122 individuals (**Table S1**). Following pre-processing, normalization, and stringent quality control (see **Materials and Methods**), principal component (PC) analysis was performed across the full dataset (comprising 531 individual DNA samples and data for a pruned set of 427,018 high-quality probes). The first PC, explaining 51.4% of the variance, clearly distinguishes between whole blood, cerebellum, and the three cortical regions (**Fig. S1**). A similar separation of these tissues is observed with the second PC, which explains 29.4% of the variance. This observation concurs with previous studies comparing whole blood and brain samples based on smaller samples and using alternative technologies for assessing DNA methylation.^23-25^ These differences between tissues are reflected in gene expression data,^26,27^ which confirm the cerebellum as being clearly distinguishable from cortical brain regions. The three cortical regions have strikingly similar DNA methylation profiles; however, while examination of further PCs, none of which explain more than 5% of the variance, starts to tease apart these regions, none of the top 20 PCs does this definitively (**Fig. S1**).

### Interindividual variation and sex make a much smaller contribution to overall variation in autosomal DNA methylation than tissue differences

**Figure 1.**
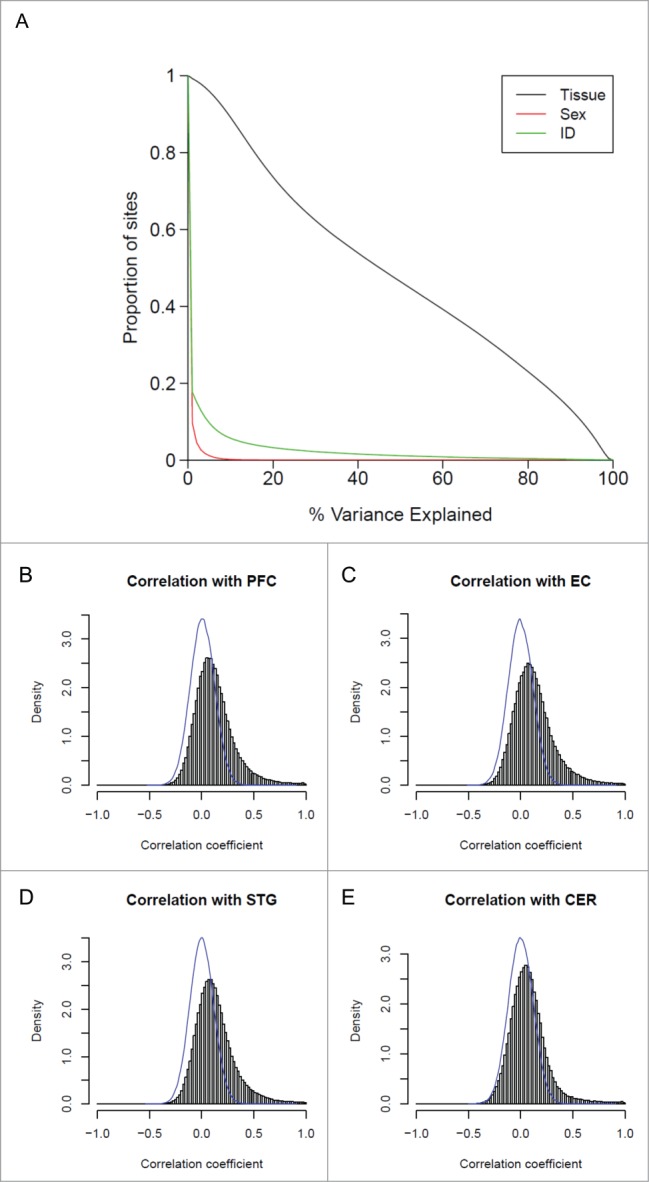
Variation in DNA methylation in whole blood is correlated with variation in the brain for a small proportion of probes. (**A**) The proportion of sites (y-axis) for which tissue (black), sex (red), or individual (green) explain a given percentage of DNA methylation variance (x-axis). (**B**) to (**E**) Histograms showing the distribution of correlation coefficients between DNA methylation in whole blood and the 4 brain regions (PFC, EC, STG and CER). For all 4 brain regions the distribution of correlation coefficients is significantly skewed to the right, with stronger correlations seen between whole blood and cortical regions than between whole blood and cerebellum.

We used linear regression models to calculate the proportion of variance in DNA methylation explained by tissue, individual, and sex. Across autosomal probes, tissue is the strongest predictor of DNA methylation (**Fig. S2**), although there is a subset of probes for which individual predicts more of the variance (5.39% of all probes) than tissue type (see **Table S2**). Across all autosomal probes passing stringent QC (n = 416,872), tissue explains > 50% of the variance in DNA methylation at 193,333 (46.4%) sites, compared to individual differences which explain > 50% of the variance at 4,669 (1.12%) sites (**Fig. 1A**). Considering only autosomal DNA methylation sites characterized as being variable in whole blood (n = 185,060, see **Materials and Methods**), these percentages increase to 66.2% and 1.61%. As expected, sex makes a strong contribution to variation observed at probes on chromosomes X and Y (n = 10,146), explaining >50% of the variance at 5,920 (58.3%) of these positions compared to tissue which explains >50% of the variance at only 1,359 (1.34%) sites. In contrast, sex makes a very small contribution to autosomal variation, explaining >50% of the variance at only 18 (4.32 × 10^−3^%) of autosomal probes (**Fig. S3**).

### Interindividual variation is correlated across tissues at a small number of sites

**Figure 2.**
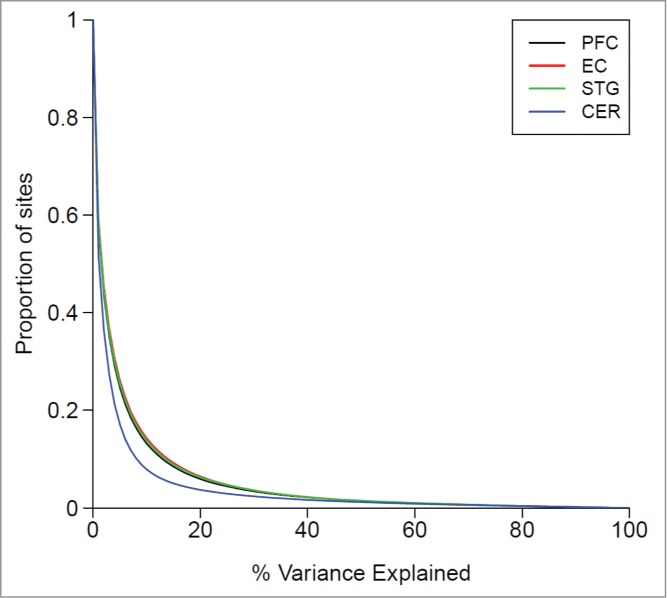
Variation in DNA methylation in whole blood as a predictor of variation in the brain. Shown is the proportion of sites (y-axis) for which variation in blood explains a certain of percentage of DNA methylation variance (x-axis) in the PFC (black), EC (red), STG (green), and CER (blue) from the same individuals.

Although overall DNA methylation profiles are clearly distinct across different brain regions and blood, driven by highly significant mean differences in DNA methylation at multiple sites across the genome,^23^ we were interested in exploring the extent to which interindividual variation detected in whole blood reflects interindividual variation in the three cortical regions and cerebellum. Focusing only on probes defined as ‘blood variable’ (n = 185,060, see **Materials and Methods**), correlation coefficients across all individuals were calculated between DNA methylation in whole blood and each of the four brain regions. Compared to the null distribution, i.e., the scenario where there is no relationship between DNA methylation in blood and brain, established by randomly permuting samples and recalculating correlations between DNA methylation in whole blood and brain across unmatched pairs, we found a modest but highly significant positive shift in the distribution of correlations for each of the four brain regions (PFC: Wilcoxon test *P* < 2.2 × 10^−308^, EC: *P* < 2.2 × 10^−308^, STG: *P* < 2.2 × 10^−308^, CER: *P* < 2.2 × 10^−308^), with a small peak highlighting a number of probes characterized by a near perfect correlation (**Fig. 1B-E****).** For the majority of probes, however, interindividual variation in DNA methylation in whole blood explains only a small amount of the variation seen in any of the brain regions (**Fig. 2**). For example, DNA methylation in whole blood is strongly correlated with levels in cerebellum (i.e., explaining >50% of the variance) for only 1.19% of “blood variable” probes, and moderately correlated (i.e., explaining >20% of the variance) with 3.68% of “blood variable” probes (see **Table S3** for corresponding values with other brain regions). Of note, the extent of interindividual correlation is significantly higher (*P* < 1.0 × 10^−308^) between whole blood and each of the three cortical regions than with the cerebellum, although the proportion of correlated probes is still low (**Table S3**). These data concur with previous small studies correlating interindividual variation in DNA methylation between tissues. Slieker et al. compared DNA methylation profiles between blood and several internal organs and reported a comparable number of sites (5,532, 3,909, 10,905, and 2,446 sites for liver, subcutaneous fat, omentum, and skeletal muscle, respectively) with a strong relationship (r > 0.8) with variation in blood.^19^ Similarly, a study comparing DNA methylation in matched blood and buccal samples found only ∼3% of 998 sites were characterized by an absolute Pearson correlation > 0.5.^20^ Density plots of DNA methylation across the probes with the strongest positive correlations (> 0.95) between blood and brain indicate that many are characterized by a clear trimodal distribution of DNA methylation **(Fig. S4**), suggesting that DNA sequence variation likely mediates much of the observed cross-tissue similarities via processes such as allele-specific DNA methylation.^28^ Many DNA methylation quantitative trait loci (mQTL) have consistent effects across tissues^29,30^; **Figure S5** shows a couple of examples where the correlated DNA methylation profiles across tissues are likely to result from mQTLs, as the distribution of DNA methylation levels cluster into distinct groups reflecting genotype, with consistent effects across tissues.

**Figure 3.**
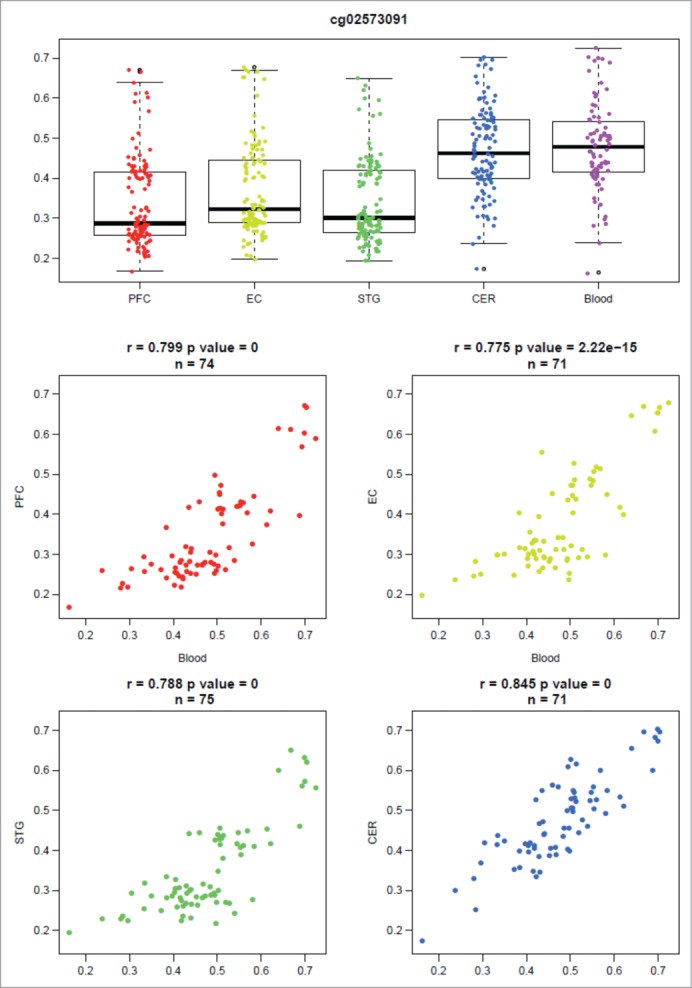
DNA methylation in whole blood significantly co-varies with that in the brain at some genomic loci. An example output of our online database (http://epigenetics.essex.ac.uk/bloodbrain/) for blood-brain correla-tions at cg26039926. Shown is a boxplot of the distribution of DNA methylation values across all individuals split by tissue and four scatterplots demonstrating the relationship between DNA methylation in whole blood and four brain regions (PFC, EC, STG, CER). At this probe there is a highly significant correlation between individual variation in whole blood and that observed in all four brain regions.

### The extent to which interindividual variation is correlated between whole blood and brain differs by brain region at some loci

Although genome-wide patterns of DNA methylation clearly distinguish between tissues, the overall extent to which interindividual variation in whole blood is correlated with that in the brain is similar across the 4 brain regions (**Figs. 3 and S6**), with a highly significant correlation of probe-wise correlations. Of note, the three cortical regions are more similar to each other in this regard than the cerebellum, indicating that there is a subset of probes where variation in whole blood predicts variation in the cortex and not the cerebellum, and vice versa (for example see **Fig. S7**). This suggests that the extent of co-variation between pairs of tissues can differ depending upon the tissues in question, and establishing a correlation between any two tissues does not imply a correlation between all tissues. Of additional interest are sites where there is a significant, but *negative*, correlation between blood and brain (see **Fig. S8** for specific examples), a phenomenon that has been reported previously for certain loci.^25^ This phenomenon is relatively rare with between 2 and 5 DNA methylation sites strongly negatively correlated (explain > 50% of the variance) between blood and cortex, and most notable between DNA methylation in whole blood and cerebellum with 19 DNA methylation sites classed as strongly negatively correlated (**Table S4**).

### Sites at which interindividual variation in DNA methylation is highly correlated between whole blood and brain are enriched in CpG-rich promoter regions

**Figure 4.**
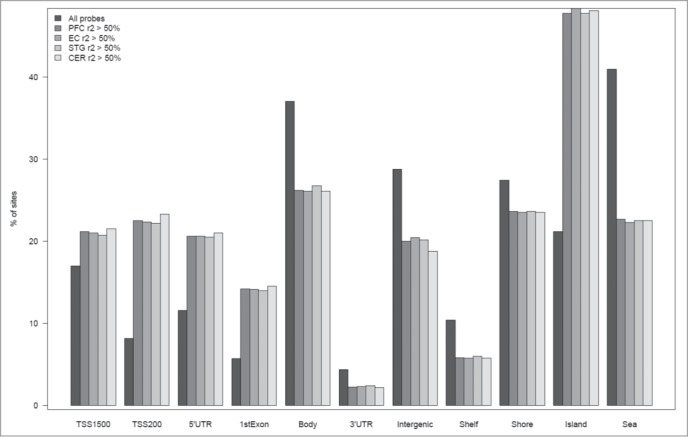
Sites at which interindividual variation correlates between whole blood and brain are enriched in specific genic features. Bar charts plotting the percentage of sites annotated to particular genic feature categories and CpG Island annotations for the full set of “blood variable” sites, in addition to the subset of sites characterized by the highest correlation (r^2^ > 50%) between blood and brain. Fisher's exact tests were used to test for either over or underrepresentation for each type of feature and are presented in **Table S2**.

Sites at which DNA methylation is strongly correlated between whole blood and brain (r^2^ > 0.5) are not equally distributed across the genome. Of note, we find a significant over-representation at loci in the vicinity of transcription start sites (PFC: *P* = 1.34 × 10^−22^, EC: *P* = 5.15 × 10^−21^, STG: *P* = 1.06 × 10^−18^, CER: *P* = 1.34 × 10^−22^), 1^st^ exon (PFC: *P* = 2.48 x 10^−175^, EC: *P* = 8.78 ×10^−174^, STG: *P* = 6.72 ×10^−171^, CER: *P* = 3.50 × 10^−172^) and 5'UTR (PFC: *P* = 2.07 × 10^−119^, EC: *P* = 5.04 × 10^−120^, STG: *P* = 2.98 × 10^−119^, CER: *P* = 2.28 × 10^−118^) and a depletion in the gene body (PFC: *P* = 6.15 × 10^−94^, EC: *P* = 4.43 ×10^−97^, STG: *P* = 2.56 × 10^−87^, CER: *P* = 1.85 × 10^−88^), 3'UTR (PFC: P = 2.92 × 10^−24^, EC: P = 1.06 × 10^−23^, STG: P = 2.02 × 10^−21^, CER: P = 4.74 × 10^−23^) and intergenic regions (PFC: P = 1.10 × 10^−72^, EC: P = 2.24 × 10^−65^, STG: P = 3.51 × 10^−71^, CER: P = 1.57 × 10^−86^) (**Fig. 4** and **Table S5**). In addition there is enrichment in CpG islands (PFC: P < 2.2 × 10^−308^, EC: P < 2.2 × 10^−308^, STG: P < 2.2 × 10^−308^, CER: P < 2.2 × 10^−308^) and depletion in open sea (PFC: P = 8.16 × 10^−262^, EC: P = 3.57 × 10^−276^, STG: P = 2.87 × 10^−273^, CER: P = 7.10 × 10^−244^) (**Fig. 4** and **Table S5**). Although these differences across genomic regions are highly significant, they may be partly biased by the relative paucity of Illumina 450K microarray probes away from CpG-rich promoter regions.

### Co-variation between tissues often occurs when absolute levels of DNA methylation are different

It is a common misconception that a similar average level of DNA methylation between two tissues at a given locus is sufficient to establish that one of these tissues may be used as a proxy for the other.^31^ In fact, for epidemiological studies that use peripheral tissues as a proxy, it is actually more important that the 2 tissues *co-vary*, regardless of their absolute DNA methylation levels. To demonstrate this point, **Table S6** lists 887 sites characterized by similar levels of DNA methylation between tissues (paired t-test *P* > 0.1) but no evidence for interindividual co-variation (r^2^ < 0.05), with specific examples shown in **Figure S9**. In contrast, **Table S7** and **Figure S10** demonstrate sites that are characterized by highly tissue-specific levels of DNA methylation (paired t-test *P* < 1.00×10^−5^) but strong evidence for interindividual co-variation (r^2^ > 0.5).

### Whole blood cannot be used as a proxy for DNA methylation sites that are only variable in the brain

**Figure 5.**
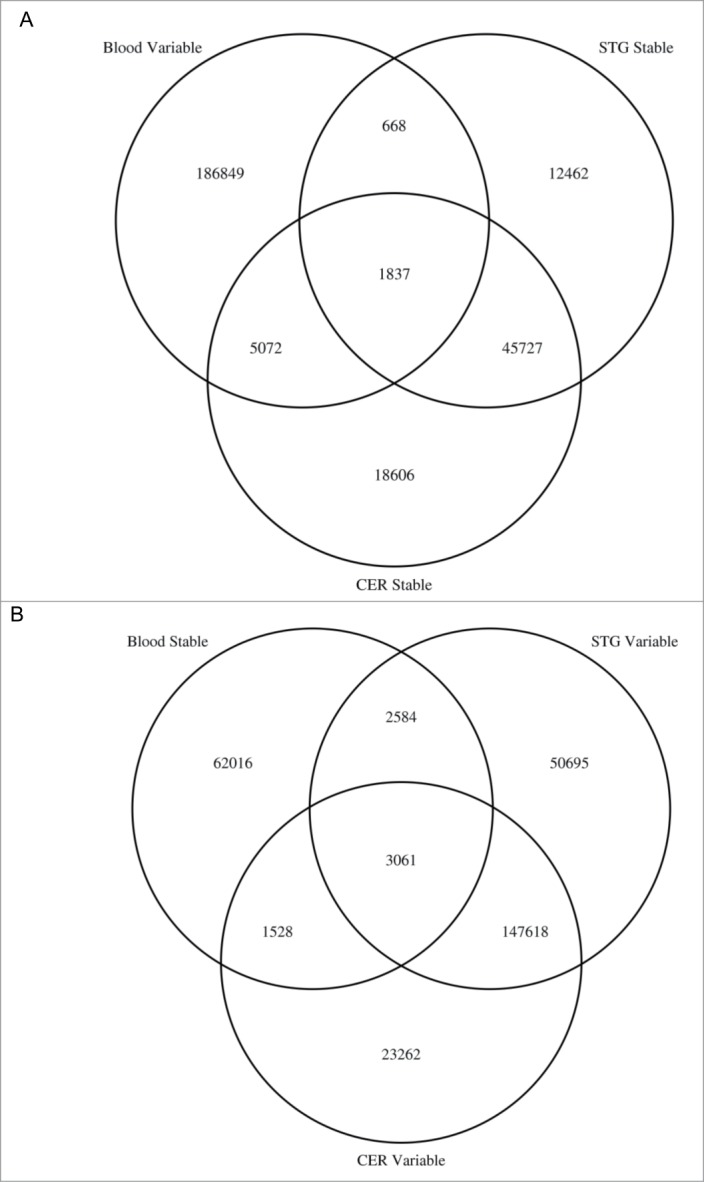
EWAS analyses of brain phenotypes using whole blood DNA may potentially miss disease associated variation and interrogate DNA methylation sites that are not actually variable in the brain. Venn diagrams showing the overlap of DNA methylation sites that are (**A**) variable in whole blood but not variable in the cortex (STG) or cerebellum and (**B**) variable in the cortex (STG) and cerebellum but not in whole blood.

One potential caveat to performing an EWAS of a neurological/psychiatric phenotype using a peripheral tissue as a proxy is that a proportion of sites are characterized by limited interindividual variation in whole blood but high levels of interindividual variation in the brain, and vice versa. We defined probes as having ‘low’ variation when the range of DNA methylation values across the total sample < 5% and ‘high’ variation when the range of DNA methylation in the middle 80^th^ percentile of samples > 5%. **Figure 5A** shows that there are 2,505 sites characterized by high interindividual variation in whole blood but not in the cortex (STG) (see **Fig. S11** and **Table S8** for corresponding data for the other cortical regions) and 6,909 sites that vary in whole blood but not in the cerebellum. Whether these sites are omitted from analyses (for example, to reduce multiple testing burden) depends upon the ultimate aim of the study being undertaken; while they may not be able to inform directly about mechanistic processes in disease, they could still represent useful biomarkers. In contrast, some sites are non-variable in whole blood but vary in the brain (see **Fig. 5B****).**

## Conclusion

Our data suggest that across the majority of the genome, an EWAS using whole blood for disorders where brain is the presumed to be the primary tissue of interest will give limited information relating to underlying pathological processes. However, there are a proportion of sites where interindividual variation is correlated between whole blood and brain, and these results do not discount the utility of using a blood-based EWAS to identify potential biomarkers of psychiatric disease phenotypes. We have developed a searchable online database (http://epigenetics.essex.ac.uk/bloodbrain/) to enable researchers to investigate the relationship between whole blood and brain for any probes on the Illumina 450K array to aid in the interpretation of EWAS analyses of brain disorders.

## Materials and Methods

### Samples

We obtained entorhinal cortex (EC), prefrontal cortex (PFC), superior temporal gyrus (STG), and cerebellum (CER) tissue from 117 individuals archived in the MRC London Neurodegenerative Disease Brain Bank (http://www.kcl.ac.uk/iop/depts/cn/research/MRC-London-Neurodegenerative-Diseases-Brain-Bank/MRC-London-Neurodegenerative-Diseases-Brain-Bank.aspx). Ethical approval for the study was provided by the NHS South East London REC 3. All samples were dissected by trained specialists, snap-frozen and stored at −80°C. Matched whole blood samples collected before death were available for 80 samples (**Table S1**) as part of the Alzheimer's Research UK funded study “Biomarkers of AD Neurodegeneration” with informed consent according to the Declaration of Helsinki (1991). Genomic DNA was isolated from ∼100 mg of each dissected brain region or ∼10 ml whole blood stored in EDTA collection tubes using a standard phenol-chloroform extraction method, and tested for degradation and purity before analysis. The samples used in this study included both neuropathologically unaffected controls and individuals with variable levels of neuropathology. More information about the specific samples can be found in Lunnon et al.^11^

### Methylomic profiling

DNA (500 ng) from each sample was sodium bisulfite-treated using the Zymo EZ 96 DNA methylation kit (Zymo Research) according to the manufacturer's standard protocol. DNA methylation was quantified using the Illumina Infinium HumanMethylation450 BeadChip (Illumina) using an Illumina HiScan System (Illumina). All samples were assigned a unique code for the purpose of the experiment and grouped by tissue and randomized with respect to other variables status to avoid batch effects, and processed in batches of 4 BeadChips. Illumina Genome Studio software was used to extract the raw signal intensities of each probe (without background correction or normalization). Raw data are downloadable from GEO with accession identifier GSE59685.

### Data pre-processing

All analyses were performed using R 3.0.2.^32^ and Bioconductor 2.13.^33^ Signal intensities were imported into R using the *methylumi* package.^34^ and transformed into β values. In order to confirm that each set of tissues derived from the same individual, initial quality control checks were performed using functions in the *methylumi* package to assess concordance between reported gender in the phenotype information and that inferred from DNA methylation sites located on the sex chromosomes. In addition, the 65 non-CpG SNP probes on the array were also used to confirm that all 4 brain regions and matched blood samples were sourced from the same individual, as their genotypes across these variants should be identical. Data was subsequently normalized in the R package *wateRmelon* using the *dasen* function as previously described.^35^ Prior to data analysis, we removed the 65 non-CpG SNP probes and probes characterized by either non-specific binding (n = 43,233) or containing common (minor allele frequency > 5%) SNPs within 10 bp of the CG or single base extension position (n = 15,261, identified from previously published lists).^36,37^ to prevent technical artifacts influencing our results. The final data set comprised 427,018 DNA methylation sites.

### Data analysis

Separate linear regression models were used to calculate the proportion of variance explained (adjusted r^2^) by a) tissue, b) individual, and c) sex, for each DNA methylation site on the array across individuals for which data from all 5 tissues passed quality control. These linear regression models took the form 
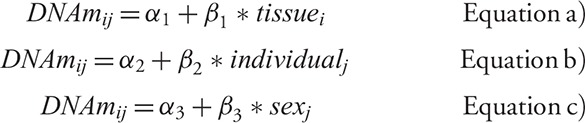


Where DNAm_ij_ is the DNA methylation value for individual j in tissue i, α is the intercept, and β the regression coefficient for each factor of interest.

A subset of “blood variable” probes was identified by calculating the DNA methylation difference between the 10^th^ and 90^th^ percentile across all samples, and selecting sites where this was > 5% (all chromosomes n = 194,426; autosomes n = 185,060). Sites characterized by overall differential DNA methylation between blood and each brain region were identified by a paired t-test of matched samples. Pairwise correlation coefficients were calculated between DNA methylation values from whole blood and each of the 4 brain regions across matched samples from linear regression models; the values were squared and multiplied by 100 to obtain the percentage of variance explained for each probe. Samples were permuted and correlations between DNA methylation in whole blood and brain were recalculated across unmatched pairs to establish the distribution in the scenario where there is no relationship between DNA methylation in blood and brain. The density curve of these simulated correlations was added to the histograms of the true correlation coefficients to represent the null distribution (**Fig. 1** and **Fig. S4**). The annotation file provided by Illumina for all probes on the array was used to classify DNA methylation sites into genomic feature and CpG island feature categories; any site with no UCSC gene annotation was classed as “intergenic." Enrichment was calculated from a 2 x 2 Fisher's exact test, comparing the number of probes with blood-brain correlation r^2^ > 0.5 annotated to each feature category to the background of all probes.

## Web Resources

A searchable database of matched blood and brain region DNA methylation data is available at http://epigenetics.essex.ac.uk/bloodbrain/. It reports the distribution of DNA methylation values in each tissue and the correlation of individual values between blood and each of the 4 brain regions for each probe on the 450K array.
