# Supplementary material for: Interindividual methylomic variation across blood, cortex, and cerebellum: implications for epigenetic studies of neurological and neuropsychiatric phenotypes
Source: Epigenetics. 2015 Oct 12;10(11):1024–32. doi: 10.1080/15592294.2015.1100786 (PMC4844197; doi:10.1080/15592294.2015.1100786)
Supplement: 1100786_Supplemental_Material.zip [file kepi-10-11-1100786-s001.zip › Supplemental Figures.pdf]

Supplementary Figures

Inter-individual methylomic variation across blood, cortex and cerebellum: implications for epigenetic studies of neurological and neuropsychiatric phenotypes.

Eilis Hannon, Katie Lunnon, Leonard Schalkwyk and Jonathan Mill

Supplementary Figure 1: The first three principal components (PCs) of genome-wide DNA methylation data clearly separates whole blood, cerebellum and cortex. PCs were calculated across all DNA methylation sites in the combined dataset of all five tissues (blood, PFC – prefrontal cortex, EC - entorhinal cortex, STG – superior temporal gyrus and CER – cerebellum). Boxplots show the distribution of PCs 1-20 grouped by tissue and identify the extent to which PCs capture variation between tissues. The first two PCs both separate blood, cerebellum and the cortical regions, while PCs 4,5 and 7 start to separate out the specific sub-cortical regions. The majority of between-tissue variation is captured in the first 11 principal components.

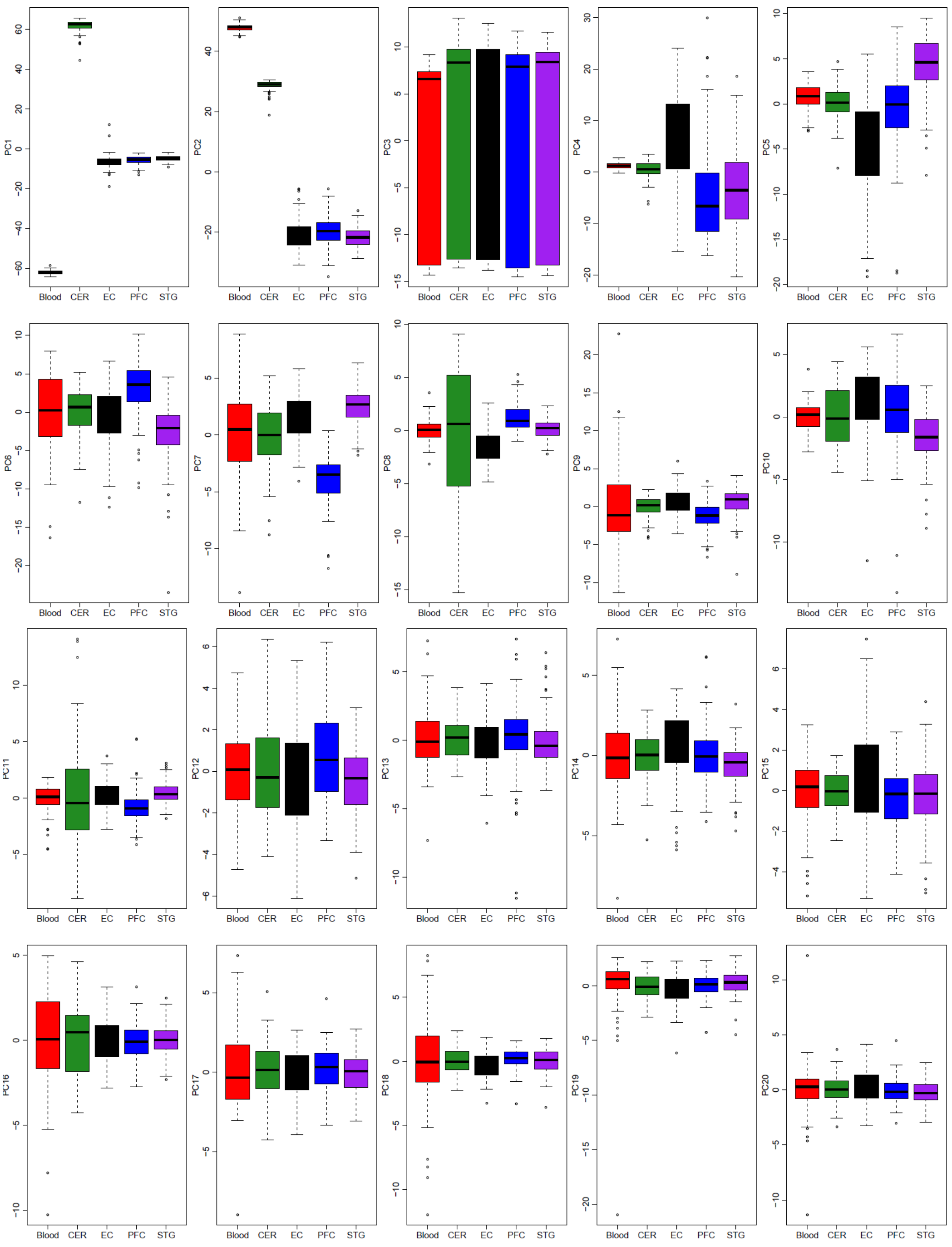

Supplementary Figure 2: Tissue type is the major determinant of DNA methylation at the majority of sites on the Illumina 450K array. Pie chart showing the proportion of sites for which the different factors (individual (ID), tissue or sex) are the best predictors of the variation in DNA methylation. The different panels represent different probe sets. A) and B) include all probes passing quality controls and C and D) only included probes that we defined as being "blood variable" (see **Materials and Methods**). A) and C) include all autosomal probes and B) and D) only include data from the sex chromosomes.

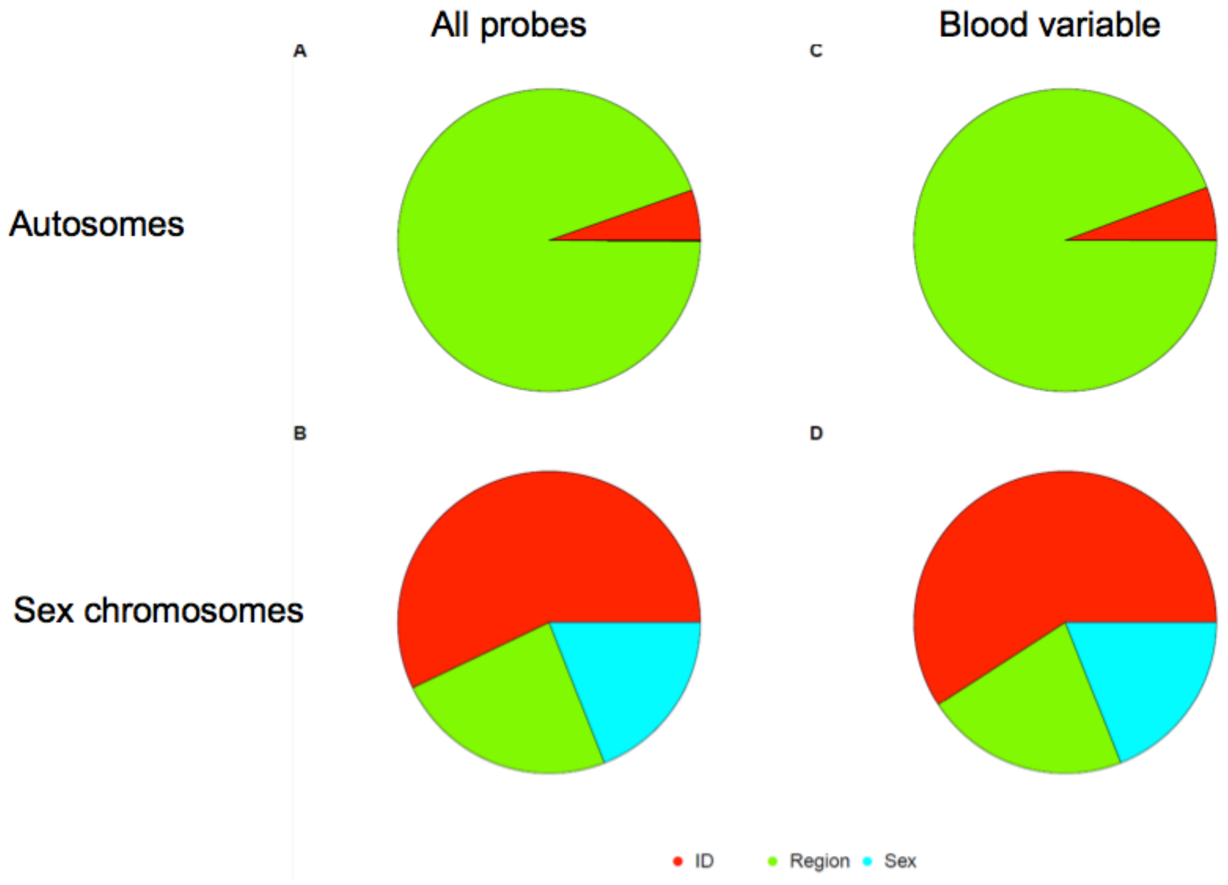

Supplementary Figure 3: For many sites tissue type strongly predicts DNA methylation level. Line graph showing the proportion of probes (y-axis) for which tissue, sex, individual or age explains a certain of percentage of DNA methylation variance (x-axis). It is evident that tissue-type contributes the most information to predicting DNA methylation. A) Sex or individual contribute little to the variation for the majority of autosomal sites but B) sex contributes more than tissue on the sex chromosomes.

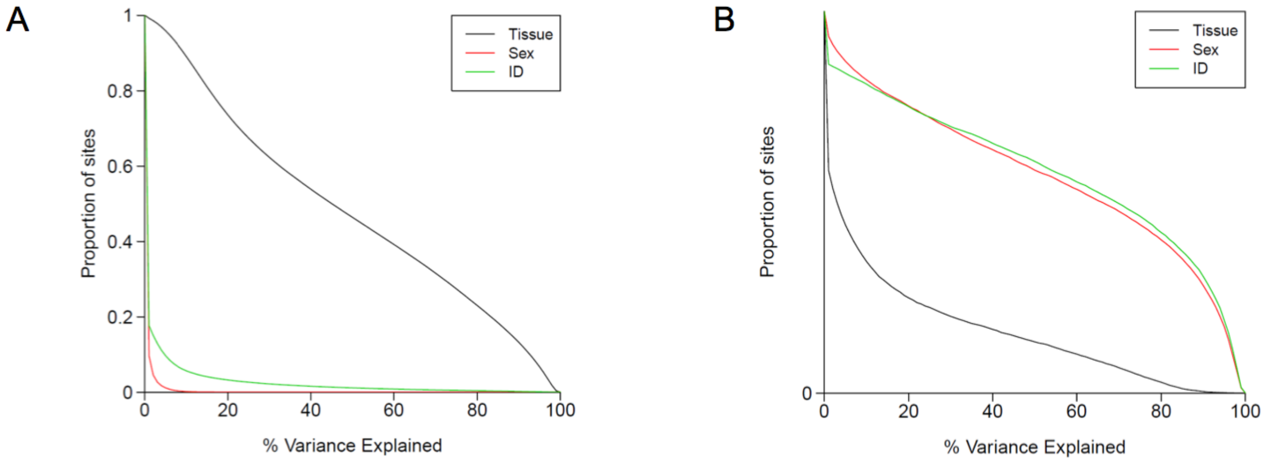

Supplementary Figure 4: Density plots of DNA methylation at sites characterized by the strongest correlation in inter-individual variation between blood and brain. These sites are demonstrate a trimodal distribution of DNA methylation values, consistent across the five tissues, indicating they are likely to be under genetic influence.

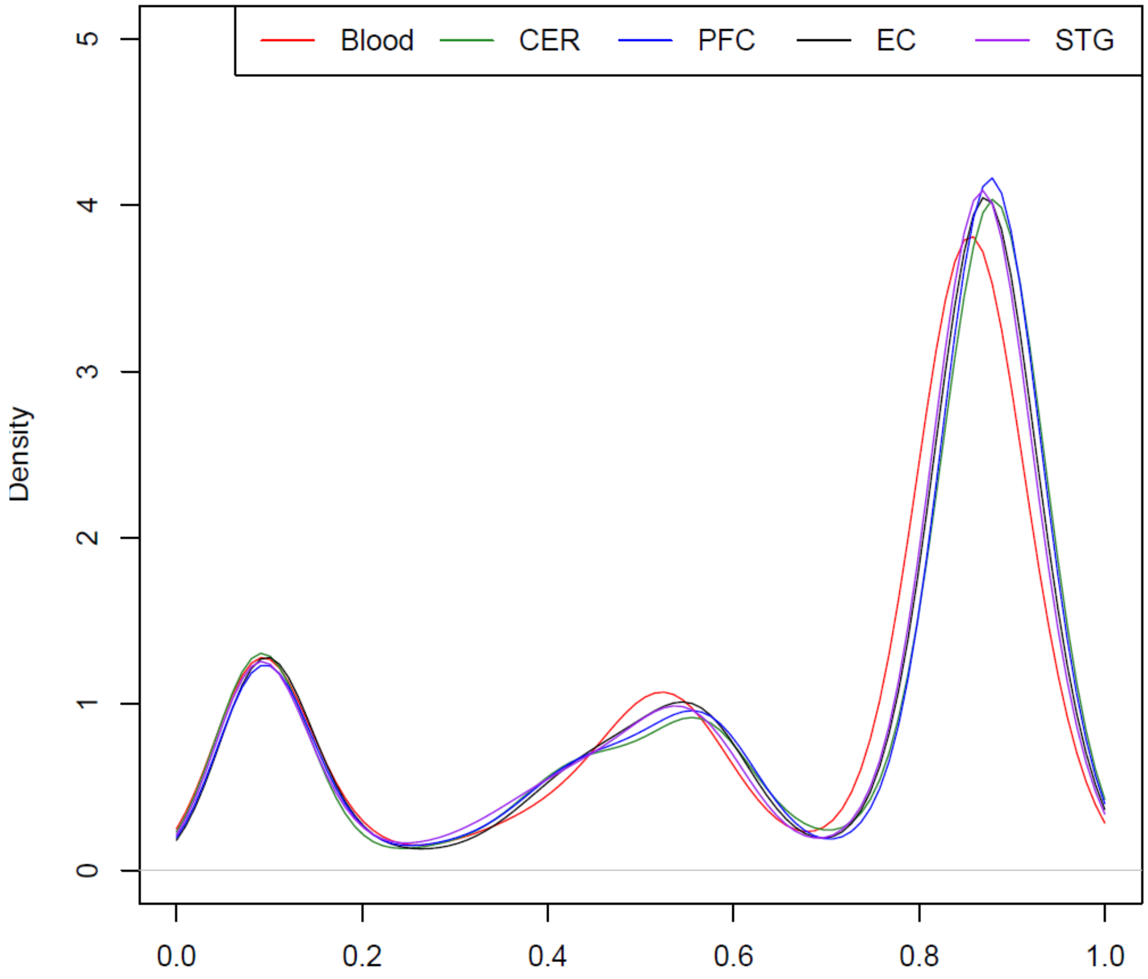

Supplementary Figure 5: Further evidence that the strongest correlations of DNA methylation across tissues is driven by genetic variation. Shown is a boxplot of the distribution of DNA methylation at cg01156747 in blood and the four brain regions. There are three clear groups, likely reflecting a genotype effect. Scatterplots of the DNA methylation values in blood against the DNA methylation values in the four brain regions shows that at this probe these genetic effects are consistent across blood and brain, and can be used to predict DNA methylation levels in the brain from blood.

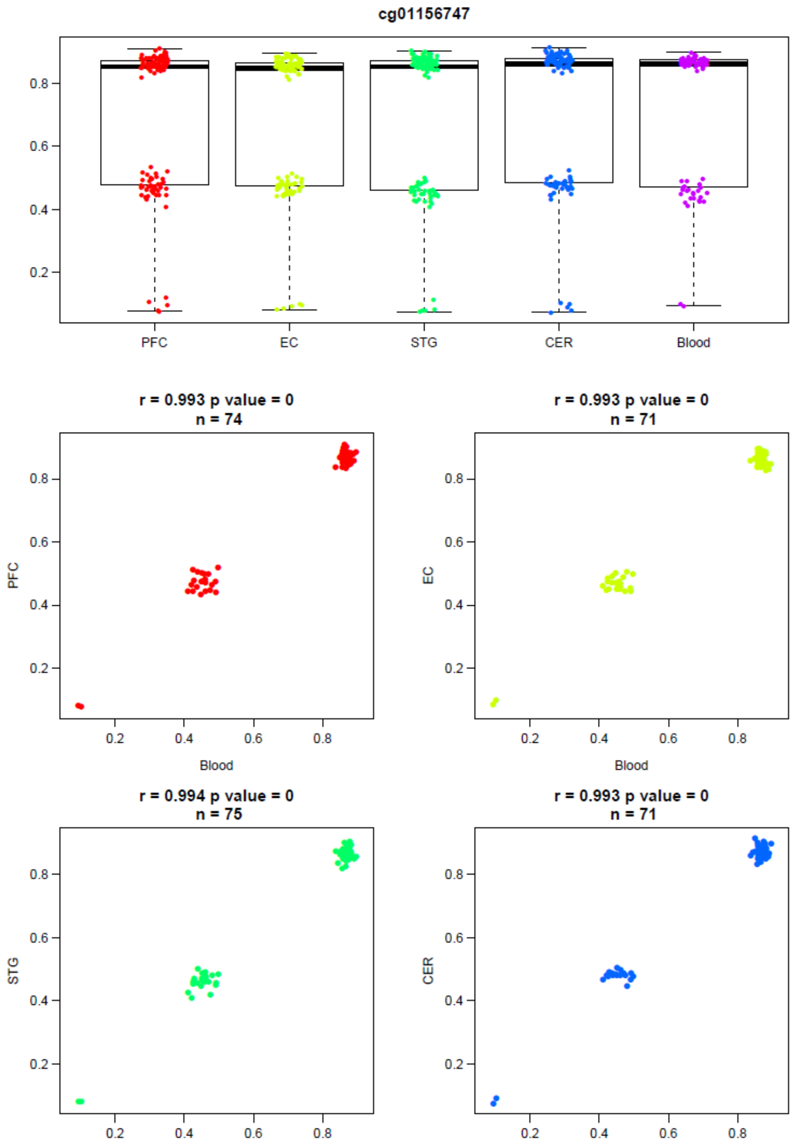

Supplementary Figure 6: Inter-individual variation in whole blood predicts inter-individual variation in different brain regions at the same sites. Comparing blood-brain correlations across individuals for all sites assessed in this study shows a very high positive correlation. This is marginally weaker, although still very strong, between CER and cortical regions, suggesting there may be a subset of probes where variation in whole blood predicts variation in CER but not the cortex and vice versa.

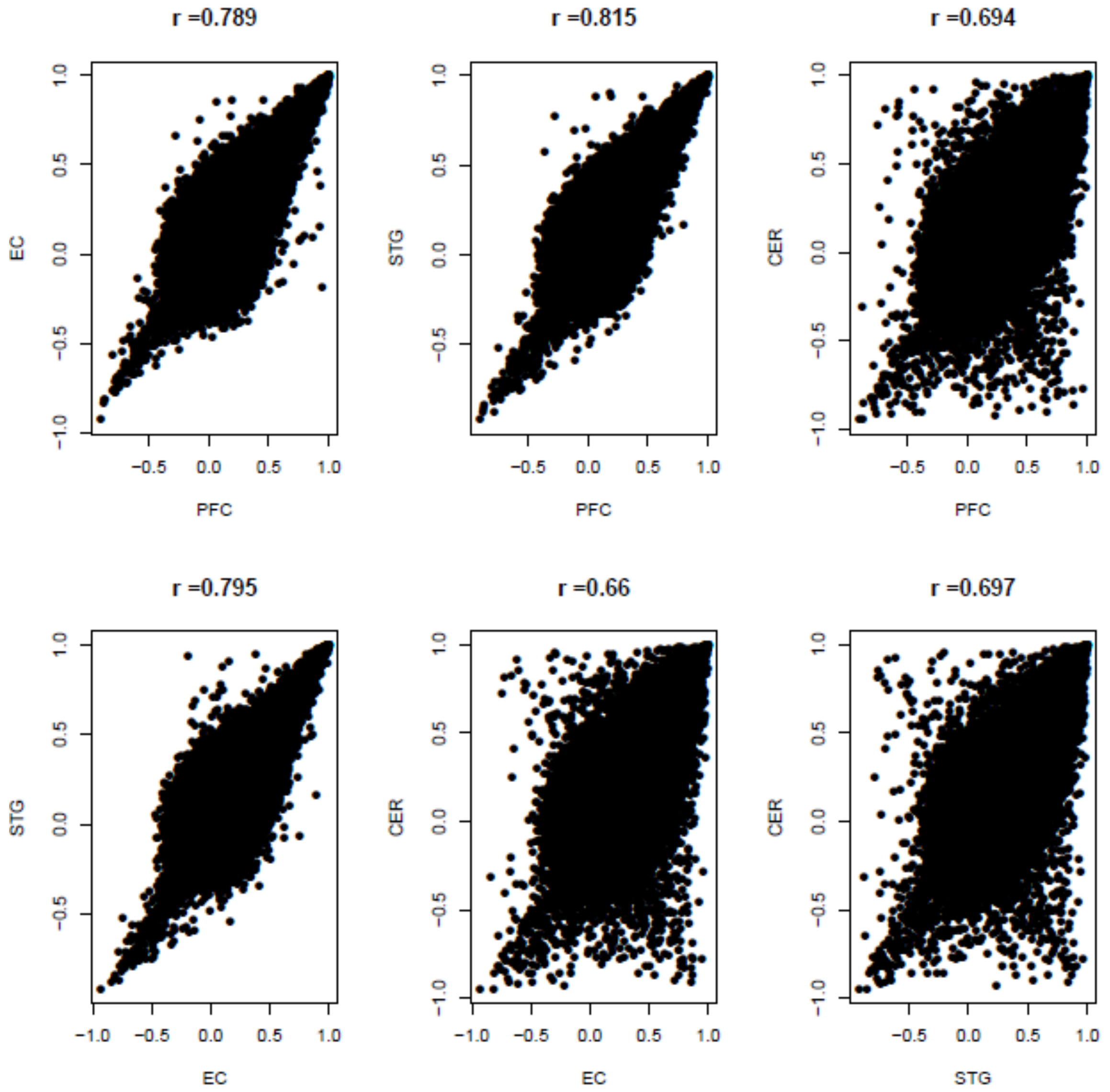

Supplementary Figure 7: Evidence of tissue-specific co-variation between whole blood and a specific brain region. A) Boxplot of the distribution of DNA methylation at cg08835221 and scatterplots of the DNA methylation values in whole blood against the DNA methylation values in the four brain regions, shows that at this probe no relationship is observed between blood and the cortical regions, but a strong positive relationship is seen between DNA methylation in blood and CER at these probes. B) Boxplot of the distribution of DNA methylation at cg06242242 in blood and four brain regions and scatterplots of the DNA methylation values in blood against the DNA methylation values in the four brain regions, shows that at this probe consistent co-variation is observed between blood and the cortical regions, but no relationship is seen between DNA methylation in blood and CER at these probes.

A

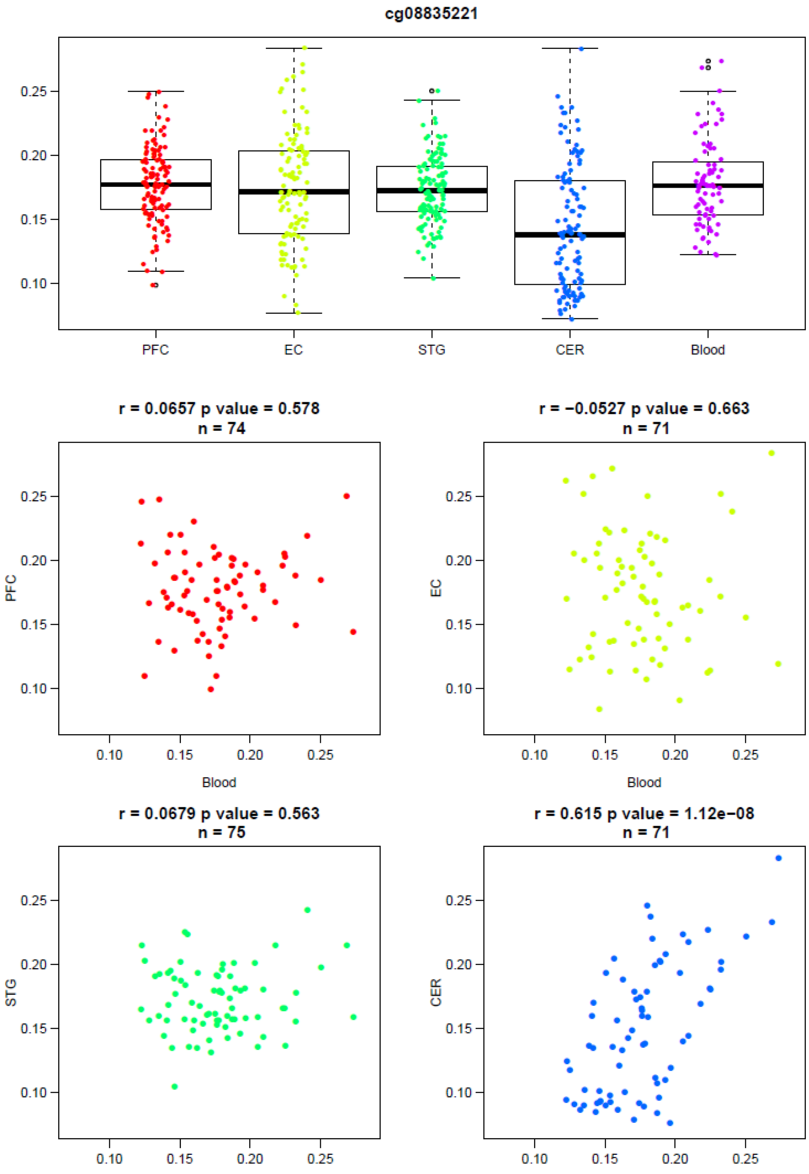

B

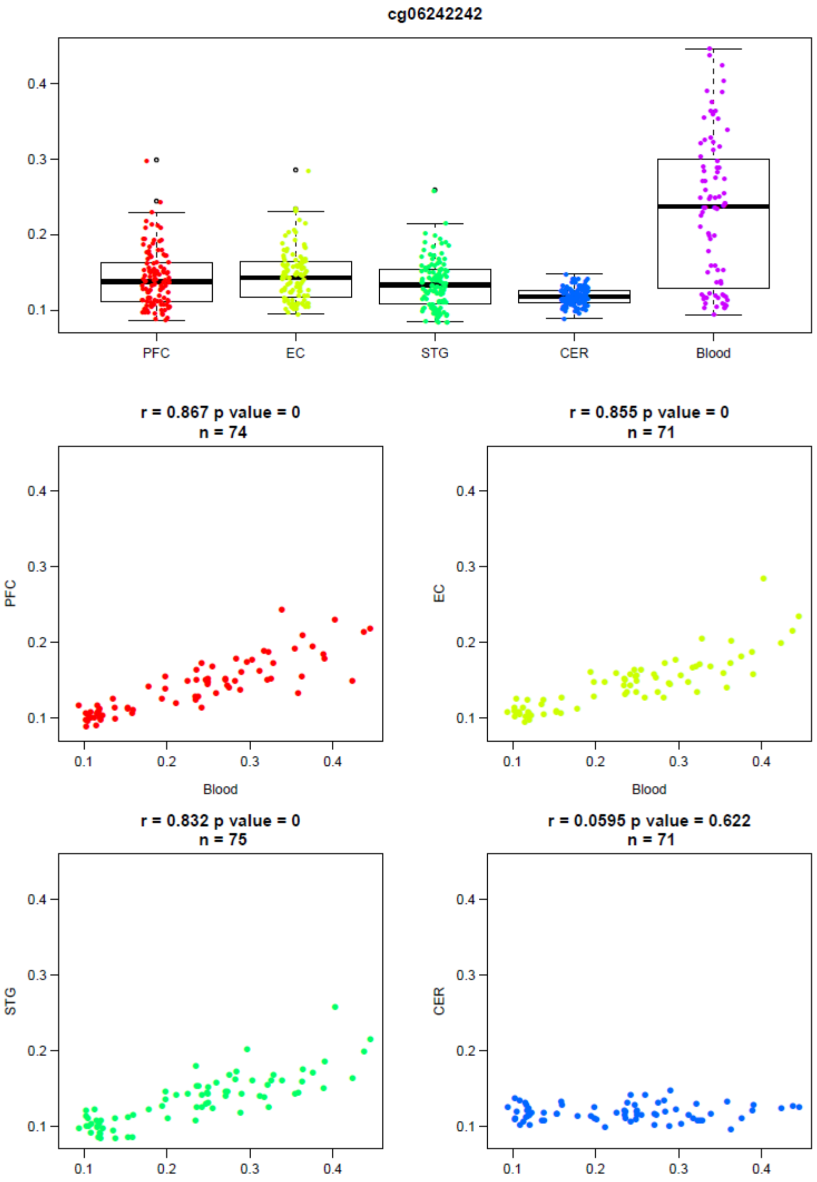
cg06242242
PFC
EC
STG
CER
Blood
r = 0.867 p value = 0
n = 74
r = 0.855 p value = 0
n = 71
r = 0.832 p value = 0
n = 75
r = 0.0595 p value = 0.622
n = 71
PFC
EC
STG
CER
Blood
Blood
0.1
0.2
0.3
0.4

Supplementary Figure 8: Some sites are characterized by a negative correlation between DNA methylation in blood and brain. Shown are two examples where inter-individual variation in whole blood is negatively correlated with inter-individual variation in the brain. A) cg20636526 and B) 12362118.

A

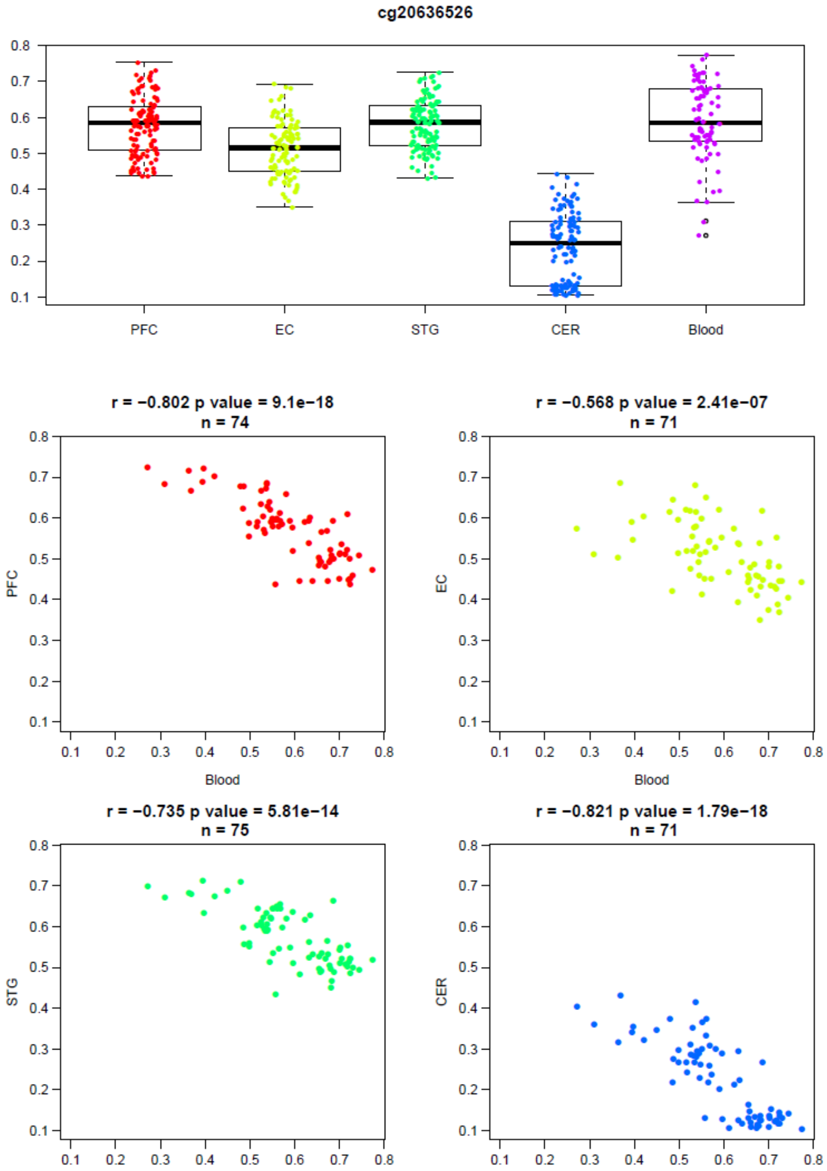

B

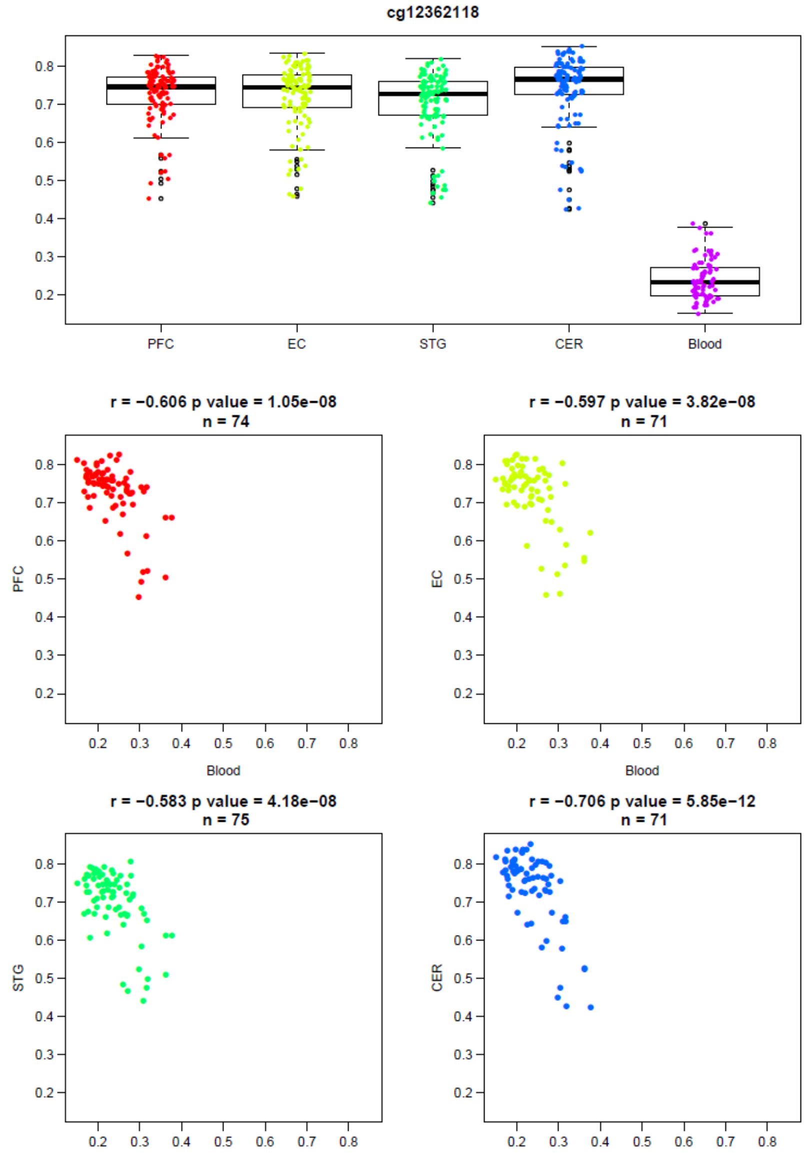

cg12362118
0.8
0.7
0.6
0.5
0.4
0.3
0.2
PFC
EC
STG
CER
Blood
r = −0.606 p value = 1.05e−08
n = 74
r = −0.597 p value = 3.82e−08
n = 71
r = −0.583 p value = 4.18e−08
n = 75
r = −0.706 p value = 5.85e−12
n = 71
0.2 0.3 0.4 0.5 0.6 0.7 0.8

Supplementary Figure 9: Example of CpG sites where the absolute DNA methylation value is similar across tissues, but inter-individual variation is not correlated. Boxplots of the distribution of DNA methylation confirm similar levels across blood and the four brain regions. Scatterplots of the DNA methylation values in blood against the DNA methylation values in the four brain regions show that at these probes there is no correlation in variation. Shown is data for A) cg00033457 and B) cg00042186.

A

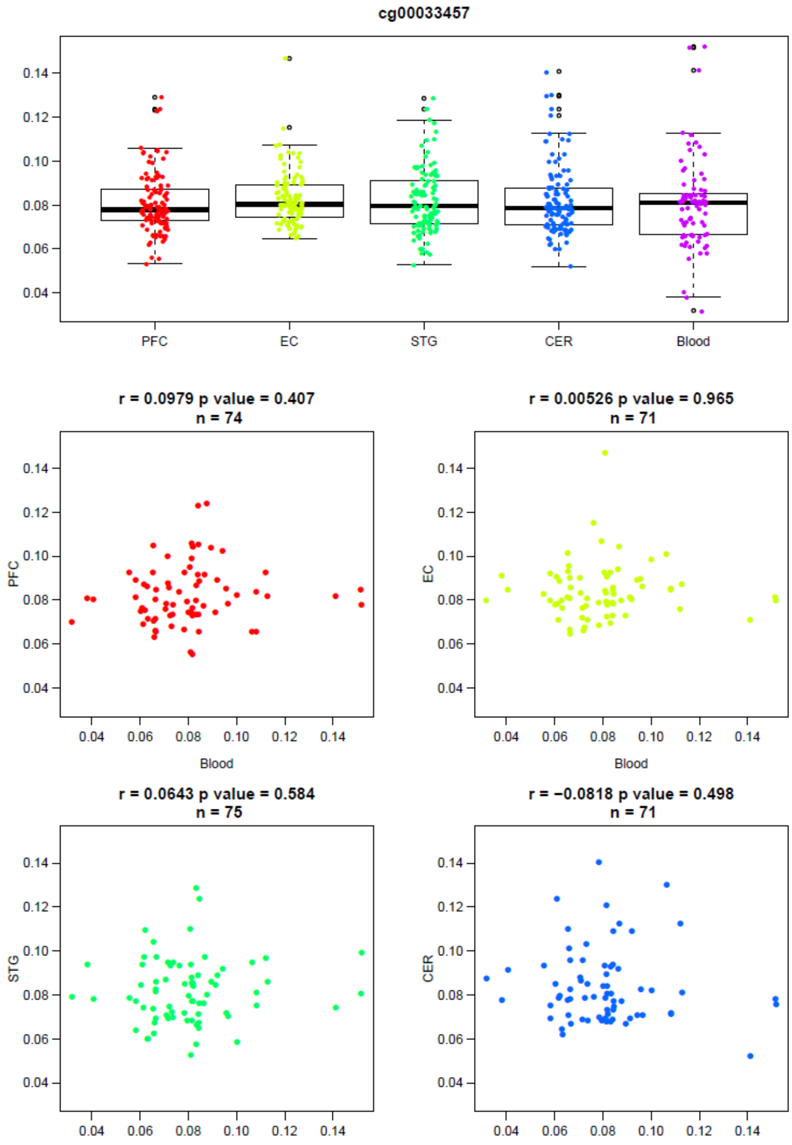

B

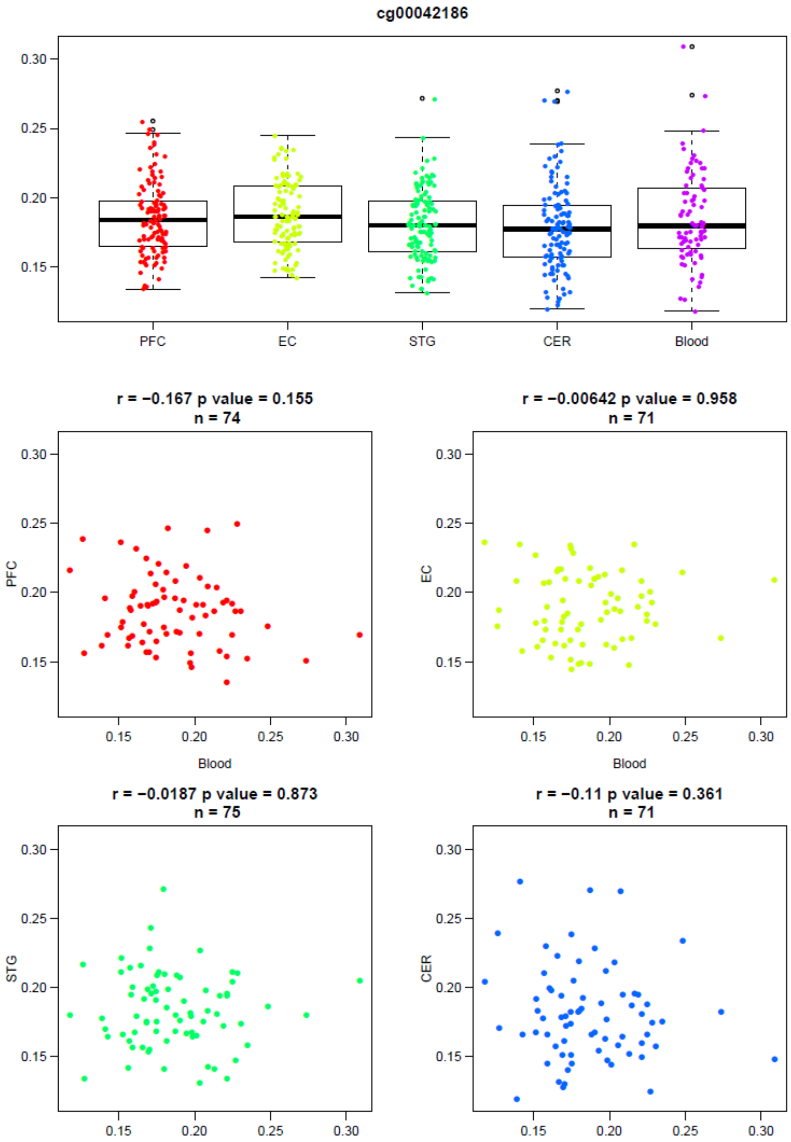

cg00042186
0.30
0.25
0.20
0.15
PFC
EC
STG
CER
Blood
r = −0.167 p value = 0.155
n = 74
r = −0.00642 p value = 0.958
n = 71
r = −0.0187 p value = 0.873
n = 75
r = −0.11 p value = 0.361
n = 71
PFC
EC
STG
CER
Blood

Supplementary Figure 10: Examples of CpG sites where absolute levels of DNA methylation are significantly different between tissues, but inter-individual variation is correlated. Boxplots of the distribution of DNA methylation confirms significant differences between blood and brain. Despite this, scatterplots of the DNA methylation values in blood against the DNA methylation values in the four brain regions show that inter-individual variation is significantly correlated across blood and brain. Shown is data for A) cg00114966 and B) cg00123128.

A

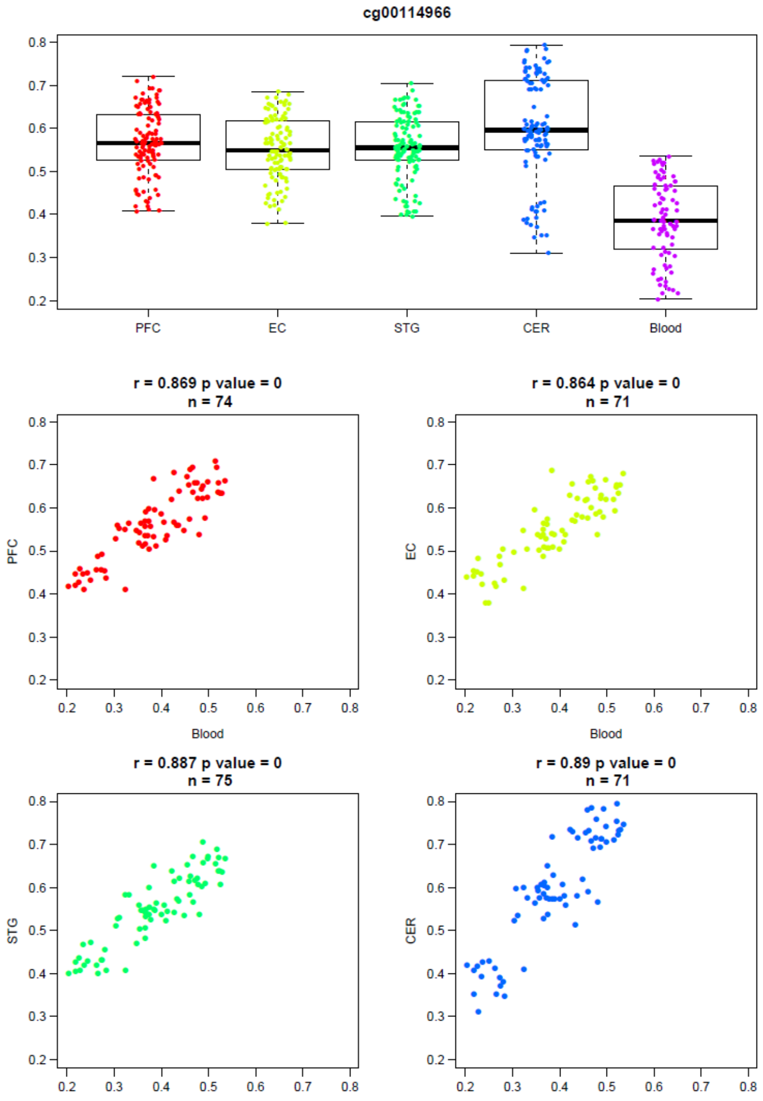

B

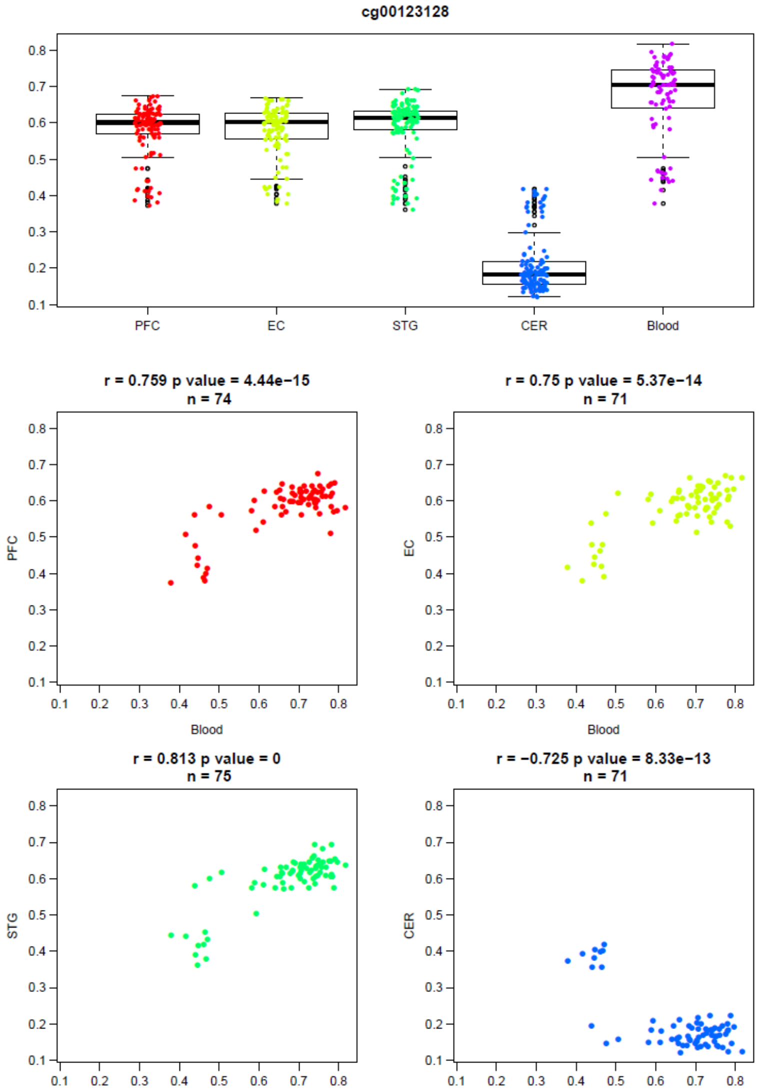

cg00123128
PFC
EC
STG
CER
Blood
r = 0.759 p value = 4.44e−15
n = 74
r = 0.75 p value = 5.37e−14
n = 71
r = 0.813 p value = 0
n = 75
r = −0.725 p value = 8.33e−13
n = 71
Blood

Supplementary Figure 11: EWAS analyses of brain-associated phenotypes using whole blood may miss potential disease associated variation and consider DNA methylation sites that do not actually vary in the brain. Venn diagrams showing the overlap of DNA methylation sites differentially variable in whole blood, cortex and cerebellum.

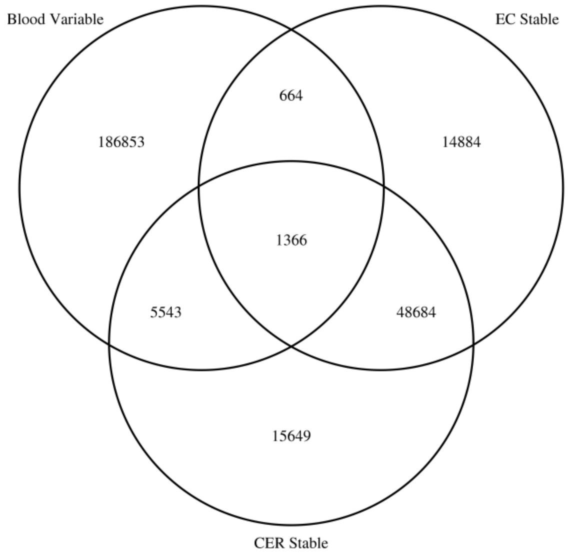

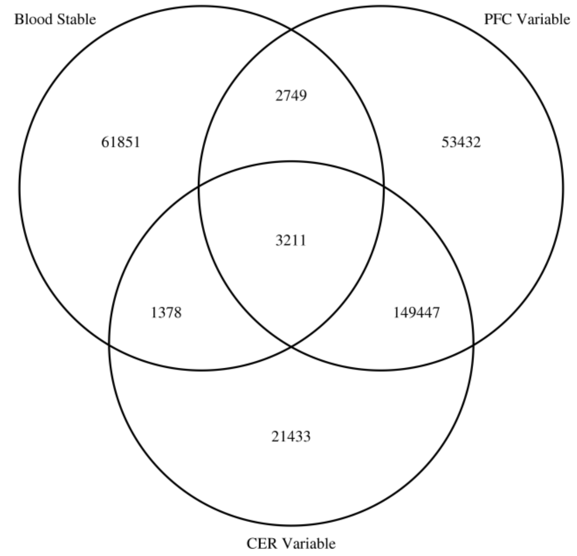

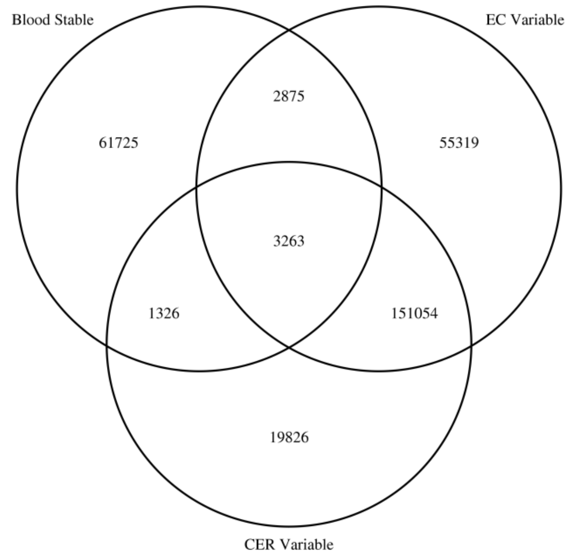
Blood Stable
EC Variable
2875
61725
55319
3263
1326
151054
19826
CER Variable

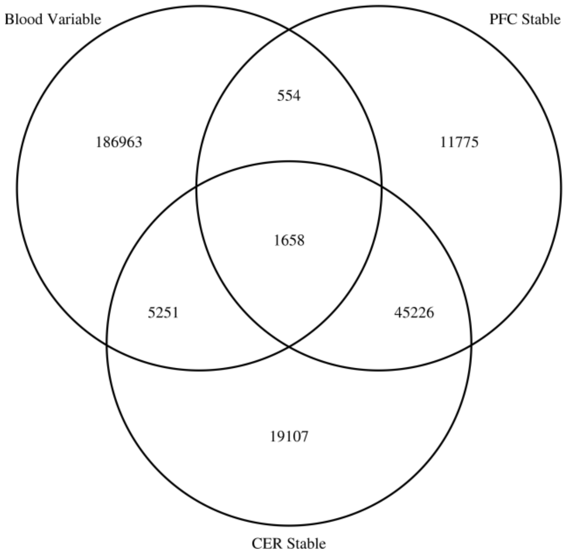
Blood Variable
PFC Stable
554
186963
11775
1658
5251
45226
19107
CER Stable
