# Supplementary material for: Interindividual methylomic variation across blood, cortex, and cerebellum: implications for epigenetic studies of neurological and neuropsychiatric phenotypes
Source: Epigenetics. 2015 Oct 12;10(11):1024–32. doi: 10.1080/15592294.2015.1100786 (PMC4844197; doi:10.1080/15592294.2015.1100786)
Supplement: 1100786_Supplemental_Material.zip [file kepi-10-11-1100786-s001.zip › Supplementary Table Captions.docx]

**Supplementary Tables**

**Supplementary Table 1:** Summary of demographic information for all samples included in this study.

**Supplementary Table 2:** Top 100 autosomal sites where individual predicts more of the variance in DNA methylation than tissue. Full table can be found at http://epigenetics.iop.kcl.ac.uk/bloodbrain/SupplementaryTables.xlsx

**Supplementary Table 3:** Counts of sites where DNA methylation in blood is correlated with brain.

**Supplementary Table 4:** Counts of sites where DNA methylation in blood is negatively correlated with brain.

**Supplementary Table 5:** Results of tests for enrichment of blood-brain correlated DNA methylation sites in specific genomic or CpG island annotation categories.

**Supplementary Table 6:** Table of 100 example sites characterized by similar levels of DNA methylation across blood and brain (paired t-test P > 0.1) but no evidence of interindividual co-variation (r^2^ < 0.05). The full table of 887 sites can be found at http://epigenetics.iop.kcl.ac.uk/bloodbrain/SupplementaryTables.xlsx

**Supplementary Table 7:** Table of 100 example sites characterized by highly tissue-specific levels of DNA methylation (paired t-test *P* < 0.00001) but strong evidence for interindividual co-variation (r^2^ > 0.5). The full table of 1,813 sites can be found at http://epigenetics.iop.kcl.ac.uk/bloodbrain/SupplementaryTables.xlsx

**Supplementary Table 8:** Table of 100 example sites, which do not vary in whole blood but do vary in at least one brain region. The full table of 7,821 sites can be found at http://epigenetics.iop.kcl.ac.uk/bloodbrain/SupplementaryTables.xlsx
