# Supplementary material for: Interindividual methylomic variation across blood, cortex, and cerebellum: implications for epigenetic studies of neurological and neuropsychiatric phenotypes
Source: Epigenetics. 2015 Oct 12;10(11):1024–32. doi: 10.1080/15592294.2015.1100786 (PMC4844197; doi:10.1080/15592294.2015.1100786)
Supplement: 1100786_Supplemental_Material.zip [file kepi-10-11-1100786-s001.zip › Table S1.docx]

|  | | **Blood** | **PFC** | **EC** | **STG** | **CER** |
| --- | --- | --- | --- | --- | --- | --- |
| **Samples** | | 80 | 114 | 108 | 117 | 112 |
| **Male** | | 27 | 38 | 35 | 40 | 38 |
| **Age at death** | |  | 55-105 | 69-105 | 40-105 | 55-105 |
| **Age at blood donation** | | 64-99 |  |  |  |  |
| **Matched blood brain samples** | **Samples** |  | 74 | 71 | 75 | 71 |
|  | **Male** |  | 26 | 23 | 26 | 26 |
|  | **Age at death** |  | 71-105 | 71-105 | 71-105 | 71-105 |
|  | **Age at blood donation** |  | 70-99 | 70-99 | 70-99 | 70-99 |
