# Supplementary material for: Interindividual methylomic variation across blood, cortex, and cerebellum: implications for epigenetic studies of neurological and neuropsychiatric phenotypes
Source: Epigenetics. 2015 Oct 12;10(11):1024–32. doi: 10.1080/15592294.2015.1100786 (PMC4844197; doi:10.1080/15592294.2015.1100786)
Supplement: 1100786_Supplemental_Material.zip [file kepi-10-11-1100786-s001.zip › Table S2.pdf]

ProbeID	Proportion of variance explained by			CHR	MAPINFO	UCSC_REFGENE_NAME	UCSC_REFGENE_GROUP	RELATION_TO_UCSC_CPG
	Region	ID	Sex					
cg04131969	-0.011791538	0.996597812	0.017604254	2	33951647	MYADML	Body	N_Shore
cg07508773	-0.0117073	0.996219795	-0.000955814	2	160143020	WDSUB1;WDSUB1;WDSUB1;WDSUB1;WDSUB1	5'UTR;5'UTR;1stExon;5'UTR;1stExon	Island
cg17717333	-0.011604511	0.994731296	-0.002748265	2	26101647	ASXL2	TSS1500	Island
cg20227766	-0.011078735	0.994128895	0.00293767	1	27998703	IFI6;IFI6;IFI6;IFI6;IFI6	1stExon;5'UTR;5'UTR;1stExon;1stExon;5'UTR	S_Shelf
cg11738485	-0.011042992	0.993576533	0.077859824	19	12877000	HOOK2;HOOK2	Body;Body	Island
cg01156747	-0.011735082	0.992991177	-0.002170644	7	120659			Island
cg14363146	-0.011788282	0.992845063	-0.002985174	9	6704178			Island
cg01818594	-0.011249365	0.992434918	-0.001020403	4	107237688	AIMP1;AIMP1;TBCK;TBCK;AIMP1;TBCK;AIMP1;TBCK	5'UTR;5'UTR;TSS1500;5'UTR;1stExon;TSS1500;5'UTR;TSS1500	
cg21438527	-0.011684532	0.991994712	-0.002800544	6	26189355	HIST1H4D	TSS200	S_Shore
cg00843105	-0.011931989	0.991456417	-0.001343572	19	44645597	ZNF234;ZNF234	TSS200;TSS200	Island
cg18093864	-0.011651972	0.991391364	0.018903257	6	8087916	EEF1E1;SCARNA27;EEF1E1	Body;TSS1500;Body	
cg05757385	-0.01027451	0.991346591	0.018513692	7	86782435	DMTF1;DMTF1;DMTF1;DMTF1;DMTF1	5'UTR;5'UTR;Body;Body;5'UTR	S_Shore
cg27210166	-0.011371886	0.990791181	-0.001713764	17	78574692	RPTOR;RPTOR	Body;Body	
cg23565757	-0.011379425	0.990291877	0.078158701	7	157646605	PTPRN2;PTPRN2;PTPRN2	Body;Body;Body	N_Shore
cg17651693	-0.011816657	0.989916734	0.001377766	19	1067423	HMHA1	1stExon	Island
cg20381404	-0.010949987	0.989698361	0.003678933	5	34008215	AMACR;AMACR;AMACR;AMACR;AMACR;AMACR;AMACR;AMACR;AMACR;AMACR;AMACR;AMACR	5'UTR;1stExon;5'UTR;1stExon;1stExon;5'UTR;1stExon;5'UTR;5'UTR;1stExon;1stExon;5'UTR	S_Shore
cg18470710	-0.010601195	0.989207242	0.024258048	15	99558719			Island
cg10536901	-0.011585925	0.988520374	0.011654032	1	108735312	SLC25A24;SLC25A24;SLC25A24	5'UTR;Body;1stExon	Island
cg12280692	-0.010289796	0.988402216	0.01967801	13	113107959			Island
cg15834841	-0.00983258	0.988120585	-0.002376734	20	5451030	LOC643406	TSS1500	
cg27361401	-0.010448097	0.98809467	0.009924387	3	32612762	DYNC1LI1	TSS1500	Island
cg15577010	-0.010909635	0.987795708	0.049306757	2	232395273	NMUR1	TSS200	Island
cg18763100	-0.01063043	0.987624505	-0.002810805	6	32917411	HLA-DMA	Body	
cg05956943	-0.009909288	0.987513157	0.064017445	8	22133375	PIWIL2;PIWIL2;PIWIL2	5'UTR;1stExon;5'UTR	S_Shore
cg10924085	-0.00986478	0.987346145	0.000676444	10	134503433	INPP5A	Body	N_Shore
cg19151808	-0.009770765	0.986769244	0.031153585	4	89619051	NAP1L5;HERC3	TSS200;Body	
cg19976628	-0.009269709	0.986302998	0.004238682	3	38033516			N_Shelf
cg13723217	-0.011109712	0.98577223	-0.003002979	17	12927897			
cg03761891	-0.007417586	0.985725311	0.019813563	3	12802190	TMEM40	TSS1500	
cg14023020	-0.011005525	0.985596597	0.028898824	8	1972405			Island
cg20349024	-0.010748927	0.985486167	0.020589928	21	38081100	SIM2;SIM2	Body;Body	Island

cg25598710	-0.009705847	0.985294123	0.011377491	2	114151353	LOC440839	Body	
cg11407598	-0.007869798	0.984621896	0.013336852	19	12753532			Island
cg01778345	-0.01130288	0.984621722	-0.002669831	1	118427435	GDAP2;GDAP2	Body;Body	
cg04784560	-0.010654574	0.984174363	0.029399628	1	221915739	DUSP10;DUSP10	TSS1500;TSS1500	Island
cg19450531	-0.011161102	0.984121571	0.002935737	3	47422572	PTPN23;PTPN23	1stExon;5'UTR	Island
cg19850149	-0.00949089	0.983753116	0.001874478	5	180397496			Island
cg06907930	-0.006264061	0.983663461	0.015895254	16	90110798	LOC100130015;GAS8;LOC100130015;GAS8	Body;3'UTR;Body;Body	
cg02621636	-0.010011035	0.983449917	0.001898649	11	60161999	MS4A14;MS4A7;MS4A7;MS4A7;MS4A14;MS4A7	TSS1500;3'UTR;3'UTR;3'UTR;TSS1500;3'UTR	
cg02836767	-0.008758724	0.983259825	0.007119315	6	152804809	SYNE1;SYNE1	Body;Body	
cg23779644	-0.007861603	0.982605675	0.006759631	6	28354168	ZSCAN12	Body	S_Shelf
cg19299952	-0.007088658	0.982412415	0.002821961	19	2078176	MOBKL2A	Body	Island
cg17527589	-0.011756454	0.982400367	0.009837788	8	39380435	ADAM3A;ADAM3A;ADAM3A	Body;Body;Body	Island
cg24233211	-0.011937485	0.982171278	-0.001744232	6	14002749			
cg13633881	-0.009310575	0.981987751	-0.002849706	9	136297879	ADAMTS13;ADAMTS13;ADAMTS13;ADAMTS13	Body;Body;Body;Body	S_Shelf
cg23281729	-0.01040421	0.981938334	0.015409383	18	78005477	PARD6G	TSS200	S_Shore
cg06373804	-0.007713526	0.981882047	0.008387566	2	242888333			
cg24668570	-0.007178312	0.981766065	-0.001729208	10	134973778	KNDC1	TSS200	Island
cg06301252	-0.010886343	0.981590399	-0.001194587	7	157504143	PTPRN2;PTPRN2;PTPRN2	Body;Body;Body	Island
cg17107246	-0.00897321	0.98156615	0.005663362	1	108735549	SLC25A24;SLC25A24	TSS200;Body	S_Shore
cg24419602	-0.010675007	0.981273052	-0.002978548	19	5510310			S_Shelf
cg09080788	-0.005979976	0.981094625	0.03826956	17	13927712	CDRT15P	TSS200	
cg23290664	-0.008879306	0.98066289	0.001049373	18	73000004	TSHZ1	Body	Island
cg06252810	-0.011097773	0.980355965	0.002171427	18	77378261			S_Shore
cg17895626	-0.010981736	0.979992712	0.02383357	17	1588142	PRPF8;PRPF8	5'UTR;1stExon	Island
cg18665209	-0.005972495	0.97973516	0.003174243	5	180397332			Island
cg08887707	-0.011682829	0.979675075	0.004857157	1	3613723	TP73;TP73;TP73;TP73	Body;Body;Body;Body	
cg27107363	-0.008380017	0.979153334	0.011948197	2	206947111	INO80D	5'UTR	N_Shelf
cg02892043	-0.002961614	0.979138483	0.021819562	2	227625209	IRS1	3'UTR	
cg18748684	-0.009530123	0.979126773	0.012647212	8	67026564			S_Shore
cg14164492	-0.007219283	0.979046402	0.042469218	1	9439003			
cg01546248	-0.007665243	0.978939166	0.000447402	22	31477006	SMTN;SMTN;SMTN	TSS1500;TSS1500;TSS1500	Island
cg01359987	-0.008652909	0.978852866	0.025667889	6	108281908			S_Shelf
cg11017226	-0.008420906	0.978286302	0.00608389	10	61666667	CCDC6;CCDC6	1stExon;5'UTR	Island
cg09616536	-0.00792843	0.978117811	0.012789217	3	64670013	ADAMTS9	Body	N_Shore
cg23905789	-0.011098852	0.977710547	0.031672408	6	32549935	HLA-DRB1	Body	N_Shore
cg06212876	-0.006618551	0.977553312	-0.001532829	21	29911755	NCRNA00161;NCRNA00161	Body;Body	

cg16344227	-0.007890349	0.977533675	0.101449855	20	56064964	HMGB1L1	TSS1500	
cg07796016	-0.009826863	0.977470684	-0.001779863	1	152779584	LCE1C	TSS1500	
cg18394648	-0.004939097	0.977311567	0.005398433	5	141538333			
cg20409752	-0.005050037	0.977177272	0.003828777	1	7122726	CAMTA1	Body	Island
cg25280720	-0.006054252	0.976345068	0.004375408	1	158532613	OR6P1	1stExon	
cg11967332	-0.01028481	0.976316243	0.014602671	1	108735228	SLC25A24;SLC25A24	Body;1stExon	Island
cg05023707	-0.006631268	0.976266965	0.073526325	8	39845127	IDO2	Body	
cg24118713	-0.006678311	0.976128624	-0.002964709	12	114088832			
cg13535098	-0.010398727	0.976003737	0.001117026	6	3000053	NQO2	TSS200	N_Shore
cg04824771	-0.010481146	0.975925984	-0.000361753	22	24372921	LOC391322	TSS200	Island
cg14451627	-0.006926595	0.975629333	0.030908787	9	115987035	SLC31A1	5'UTR	S_Shelf
cg00506198	-0.009300452	0.975581295	0.039725415	19	17918523	B3GNT3	5'UTR	N_Shore
cg18110333	-0.009412672	0.975574822	-0.002901125	6	292329	DUSP22;DUSP22	1stExon;5'UTR	Island
cg04482597	-0.009517994	0.975567072	0.025266217	9	37486041	POLR1E;POLR1E	1stExon;5'UTR	Island
cg13830619	-0.011910103	0.975517285	0.021272426	12	9555480			
cg07423892	-0.00982903	0.975500215	0.009084162	1	236956644			N_Shore
cg08066154	-0.009366435	0.975402334	0.009706208	12	50459310	ACCN2;ACCN2	Body;Body	
cg14638919	-0.002170241	0.975311544	0.056783544	9	138065530			N_Shelf
cg24617313	-0.010418712	0.97524143	0.053920208	20	57427146	GNAS;GNAS;GNAS;GNASAS	TSS1500;TSS1500;3'UTR;TSS1500	N_Shore
cg21445911	-0.001068031	0.97519633	-0.002928815	22	43741137			S_Shore
cg01516881	-0.011511248	0.975166122	-0.002921965	6	292596	DUSP22	Body	Island
cg03395511	-0.009668677	0.974831838	-0.002586159	6	291903	DUSP22	TSS200	N_Shore
cg04206665	-0.008972471	0.974802293	0.102462216	1	1113501	TTLL10	5'UTR	N_Shelf
cg27129755	-0.00850242	0.974445118	0.005054977	5	170210586	GABRP	TSS200	
cg27157669	-0.008052273	0.974162303	0.041396491	12	27936825	KLHDC5	Body	S_Shelf
cg18135502	-0.009993331	0.974130271	-0.001714278	10	126245651	LHPP;LHPP	Body;Body	
cg17266515	-0.006776863	0.974116437	-0.002583987	2	234627262	UGT1A10;UGT1A6;UGT1A8;UGT1A6;UGT1A9;UGT1A4;UGT1A7;UGT1A5	Body;Body;Body;Body;Body;TSS200;Body;Body	
cg05926478	-0.006754039	0.97399498	-0.001669007	4	151174724	DCLK2;DCLK2	Body;Body	N_Shelf
cg15115757	-0.008778535	0.973694322	-0.001750302	6	32795627	TAP2;TAP2	3'UTR;Body	
cg23804921	-0.001184313	0.973597973	-0.001847181	15	30861172			N_Shelf
cg05380919	-0.007459507	0.973335967	0.002040022	22	24376252	GSTT1	3'UTR	S_Shelf
cg09370299	-0.010595067	0.973180246	0.009594036	2	136577346	LCT	Body	
cg01238044	-0.004524382	0.973161369	0.001656764	22	24384105	GSTT1	Body	N_Shore
