# Supplementary material for: Interindividual methylomic variation across blood, cortex, and cerebellum: implications for epigenetic studies of neurological and neuropsychiatric phenotypes
Source: Epigenetics. 2015 Oct 12;10(11):1024–32. doi: 10.1080/15592294.2015.1100786 (PMC4844197; doi:10.1080/15592294.2015.1100786)
Supplement: 1100786_Supplemental_Material.zip [file kepi-10-11-1100786-s001.zip › Table S3.docx]

| **Probes where interindividual variation in blood correlated with interindividual variation in** | **Strongly predictive (> 50% variance)** | **Moderately predictive (> 20% of the variance)** |
| --- | --- | --- |
| PFC | 2631 (1.42%) | 10900 (5.89%) |
| EC | 2698 (1.46%) | 11946 (6.46%) |
| STG | 2764 (1.49%) | 11574 (6.25%) |
| CER | 2197 (1.19%) | 6810 (3.68%) |
