# Supplementary material for: Interindividual methylomic variation across blood, cortex, and cerebellum: implications for epigenetic studies of neurological and neuropsychiatric phenotypes
Source: Epigenetics. 2015 Oct 12;10(11):1024–32. doi: 10.1080/15592294.2015.1100786 (PMC4844197; doi:10.1080/15592294.2015.1100786)
Supplement: 1100786_Supplemental_Material.zip [file kepi-10-11-1100786-s001.zip › Table S4.pdf]

Interindividual variation negatively correlated with interindividual variation in	Strongly predictive (> 50% variance)
PFC	4
EC	2
STG	5
CER	19

Moderately predictive (> 20% of the variance)
29
28
37
129
