# Supplementary material for: Interindividual methylomic variation across blood, cortex, and cerebellum: implications for epigenetic studies of neurological and neuropsychiatric phenotypes
Source: Epigenetics. 2015 Oct 12;10(11):1024–32. doi: 10.1080/15592294.2015.1100786 (PMC4844197; doi:10.1080/15592294.2015.1100786)
Supplement: 1100786_Supplemental_Material.zip [file kepi-10-11-1100786-s001.zip › Table S5.pdf]

		All		PFC				EC				STG				CER			
		n	%	n	%	P value	OR	n	%	P value	OR	n	%	P value	OR	n	%	P value	OR
**Total**		194426		7747				7806				7932				7096			
**Genic Annotation**	**TSS1500**	33072	17.01	1644	21.22	1.34E-22	1.33	1642	21.04	5.15E-21	1.32	1646	20.75	1.06E-18	1.29	1528	21.53	1.34E-23	1.36
	**TSS200**	15857	8.16	1747	22.55	0	3.56	1746	22.37	0	3.52	1760	22.19	0	3.49	1655	23.32	0	3.71
	**5'UTR**	22564	11.61	1598	20.63	2.07E-119	2.05	1609	20.61	5.04E-120	2.05	1626	20.5	2.98E-119	2.04	1493	21.04	2.28E-118	2.1
	**1stExon**	11093	5.71	1101	14.21	2.48E-175	2.93	1103	14.13	8.78E-174	2.91	1108	13.97	6.72E-171	2.87	1034	14.57	3.50E-172	3.01
	**Body**	72002	37.03	2033	26.24	6.15E-94	0.59	2038	26.11	4.43E-97	0.59	2122	26.75	2.56E-87	0.61	1851	26.09	1.85E-88	0.59
	**3'UTR**	8527	4.39	175	2.26	2.92E-24	0.49	179	2.29	1.06E-23	0.5	191	2.41	2.02E-21	0.53	158	2.23	4.74E-23	0.49
	**Intergenic**	55981	28.79	1550	20.01	1.10E-72	0.61	1599	20.48	2.24E-65	0.63	1602	20.2	3.51E-71	0.61	1334	18.8	1.57E-86	0.56
**CpG Island Annotation**	**Shelf**	20232	10.41	450	5.81	5.00E-48	0.52	449	5.75	1.18E-49	0.51	475	5.99	3.37E-45	0.54	411	5.79	2.29E-44	0.52
	**Shore**	53334	27.43	1834	23.67	1.88E-14	0.81	1839	23.56	2.21E-15	0.81	1876	23.65	6.00E-15	0.81	1670	23.53	3.07E-14	0.81
	**Island**	41192	21.19	3703	47.8	0	3.64	3776	48.37	0	3.74	3792	47.81	0	3.65	3415	48.13	0	3.67
	**Sea**	79668	40.98	1760	22.72	8.16E-262	0.41	1742	22.32	3.57E-276	0.4	1789	22.55	2.87E-273	0.41	1600	22.55	7.10E-244	0.41
