# Supplementary material for: Interindividual methylomic variation across blood, cortex, and cerebellum: implications for epigenetic studies of neurological and neuropsychiatric phenotypes
Source: Epigenetics. 2015 Oct 12;10(11):1024–32. doi: 10.1080/15592294.2015.1100786 (PMC4844197; doi:10.1080/15592294.2015.1100786)
Supplement: 1100786_Supplemental_Material.zip [file kepi-10-11-1100786-s001.zip › Table S6.pdf]

ProbeID	Blood PFC correlation		Blood EC correlation		Blood STG correlation		Blood CER correlation		Difference between blood and PFC		Difference between blood and EC		Difference between blood and STG		Difference between blood and CER		CHR	MAPINFO	UCSC_REFGENE_NAME	RELATION_TO_UCSC_CPG_ISLAND
	r	p value	r	p value	r	p value	r	p value	Mean diff.	P value	Mean diff.	P value	Mean diff.	P value	Mean diff.	P value				
cg00005543	0.004	0.973	0.097	0.42	-0.075	0.522	-0.01	0.936	-0.001	0.563	0.001	0.282	-0.002	0.131	0.002	0.14	6	170151632	TCTE3;C6orf70;TCTE3	Island
cg00017441	0.033	0.78	0.007	0.955	-0.057	0.625	0.064	0.594	0	0.816	0	0.905	-0.001	0.399	0	0.95	19	18220973	MAST3	Island
cg00031967	-0.191	0.102	0.11	0.363	0.037	0.755	-0.029	0.809	0.001	0.186	0	0.926	0	0.849	0.001	0.311	7	43769114	C7orf44	Island
cg00033457	0.098	0.407	0.005	0.965	0.064	0.584	-0.082	0.498	0.002	0.392	0.004	0.177	0.002	0.543	0.003	0.379	8	74884507	TCEB1	Island
cg00039044	0.021	0.859	-0.015	0.904	0.008	0.948	0.073	0.543	0.001	0.313	0.001	0.523	0	0.754	0.002	0.241	4	83351858	ENOPH1;ENOPH1;HNRPDL;HNRPDL	Island
cg00042186	-0.167	0.155	-0.006	0.958	-0.019	0.873	-0.11	0.361	0	0.946	0.003	0.486	-0.003	0.583	-0.006	0.343	2	47748839	KCNK12	Island
cg00049286	0.111	0.345	0.144	0.231	0.168	0.151	0.111	0.356	0.002	0.146	0	0.963	-0.001	0.31	0.001	0.25	2	120436657	TMEM177;TMEM177;TMEM177	Island
cg00100012	-0.071	0.548	0.179	0.134	-0.2	0.085	-0.214	0.072	-0.001	0.592	0.003	0.222	-0.003	0.237	0.004	0.264	20	37302983		Island
cg00106591	0.059	0.618	0.176	0.141	0.066	0.575	0.044	0.717	0.002	0.706	-0.002	0.687	-0.006	0.267	0.007	0.206	16	34209017		Island
cg00117463	0.017	0.886	0.144	0.231	0.127	0.277	-0.02	0.866	0.001	0.538	-0.001	0.555	-0.001	0.328	-0.001	0.497	6	151815241	C6orf97;C6orf97	Island
cg00291003	0.029	0.807	0.117	0.332	-0.027	0.82	0.045	0.711	-0.001	0.608	0.001	0.622	-0.003	0.252	0.003	0.23	10	75541506	CHCHD1	N_Shore
cg00321838	0.108	0.359	0.048	0.689	0.041	0.727	-0.073	0.546	0.01	0.28	0.011	0.236	0.008	0.27	-0.006	0.423	8	7885901		Island
cg00378717	0.21	0.073	0.057	0.638	0.082	0.485	0.056	0.643	0.003	0.509	0.007	0.241	0.003	0.576	-0.003	0.568	23	20160007	EIF1AX	Island
cg00394494	0.049	0.676	0.1	0.404	-0.139	0.234	0.095	0.432	-0.002	0.302	0.001	0.485	-0.001	0.754	-0.001	0.531	2	167232645	SCN9A	Island
cg00395579	-0.147	0.212	0.021	0.861	0.056	0.631	-0.019	0.875	-0.003	0.241	0	0.998	-0.002	0.241	0	0.988	8	103243598	RRM2B	
cg00397324	0.036	0.759	0.046	0.706	-0.004	0.973	0.054	0.656	0	0.979	0.002	0.651	0	0.951	-0.005	0.21	21	42798594	MX1;MX1	Island
cg00467202	0.011	0.927	0.17	0.157	-0.116	0.32	0.089	0.458	0.001	0.25	0.001	0.361	-0.001	0.307	-0.001	0.11	16	30759573	PHKG2	Island
cg00468144	0.179	0.127	0.036	0.767	-0.103	0.377	0.163	0.174	0	0.891	-0.001	0.508	-0.001	0.568	0.001	0.245	1	113008976	WNT2B	Island
cg00508499	0.056	0.635	0.02	0.867	0.183	0.116	-0.218	0.067	-0.002	0.328	0.003	0.175	0	1	-0.003	0.299	22	38054505	PDXP	Island
cg00522679	0.094	0.425	0.163	0.174	-0.05	0.667	-0.021	0.86	0.001	0.563	-0.001	0.393	-0.001	0.123	-0.001	0.161	11	130083160		S_Shelf
cg00533084	0.042	0.723	-0.004	0.971	-0.151	0.197	-0.089	0.463	-0.002	0.17	0.002	0.303	-0.002	0.277	0.001	0.705	1	156698170	C1orf66;ISG20L2;C1orf66	Island
cg00542041	0.177	0.132	-0.088	0.467	-0.1	0.394	0.036	0.763	0.001	0.295	0.001	0.448	-0.002	0.104	-0.001	0.223	1	1272559	DVL1	Island
cg00591160	0.034	0.772	-0.069	0.567	-0.137	0.24	0.015	0.899	-0.007	0.197	0.007	0.284	-0.004	0.568	-0.006	0.341	12	6772270	ING4;ING4;ING4;ING4;ING4;ING4;ING4;ING4;ING4;ING4;ING4;ING4	Island
cg00598261	0.093	0.429	-0.101	0.404	0.118	0.311	0.212	0.075	-0.002	0.129	0	0.944	0	0.74	-0.001	0.689	6	105307892	HACE1	Island

cg00608467	-0.169	0.15	0.051	0.673	0.009	0.941	-0.209	0.08	0	0.727	0	0.835	-0.001	0.211	-0.001	0.413	22	46546191	PPARA;PPARA	Island
cg00616447	0.192	0.102	0.113	0.35	0.216	0.063	0.072	0.549	-0.005	0.13	0.001	0.634	0	0.937	-0.001	0.628	15	41694635	NDUFAF1;NDUFAF1	Island
cg00705878	-0.093	0.433	-0.131	0.275	0.079	0.498	-0.013	0.911	0.001	0.338	0.001	0.507	0	0.786	0.001	0.29	3	142720294	SR140	Island
cg00731404	0.221	0.059	0.009	0.939	0.026	0.826	0.065	0.589	-0.001	0.388	0	0.918	-0.001	0.27	0.001	0.282	13	28024444	MTIF3;MTIF3;MTIF3;MTIF3	Island
cg00754232	-0.11	0.349	-0.035	0.771	0.146	0.21	0.105	0.386	0	0.553	0.001	0.242	-0.001	0.117	-0.001	0.165	7	97881723	TECPR1	Island
cg00817396	0.09	0.444	0.109	0.363	0.149	0.201	0.047	0.697	0.003	0.152	0.003	0.183	-0.002	0.175	0	0.801	13	21347357	N6AMT2	N_Shore
cg00826921	-0.061	0.606	-0.067	0.578	0.086	0.463	-0.051	0.675	0.002	0.386	0.002	0.128	-0.002	0.134	-0.001	0.403	3	143692146	C3orf58;C3orf58	Island
cg00837649	0.092	0.437	0.135	0.262	0.01	0.929	-0.134	0.265	-0.002	0.466	-0.002	0.436	0.002	0.509	-0.004	0.231	3	67705228	SUCLG2	S_Shore
cg00842351	-0.141	0.229	-0.145	0.226	0.18	0.122	-0.023	0.851	0.003	0.318	0.002	0.423	0	0.933	0.001	0.749	9	71789653	TJP2;TJP2;TJP2;TJP2	S_Shore
cg00930706	-0.012	0.92	0.147	0.221	-0.143	0.222	0.2	0.094	0.001	0.177	0.001	0.53	0.001	0.58	0.001	0.523	16	88752925	MGC23284;SNAI3	Island
cg00971834	0.046	0.695	-0.093	0.439	-0.047	0.688	0.054	0.658	0.001	0.569	0.001	0.254	0.001	0.581	-0.001	0.637	10	71561574	COL13A1;COL13A1;COL13A1;COL13A1;COL13A1;COL13A1;COL13A1;COL13A1;COL13A1;COL13A1;COL13A1;COL13A1;COL13A1;COL13A1;COL13A1;COL13A1;COL13A1;COL13A1;COL13A1;COL13A1	Island
cg00975418	-0.003	0.978	-0.111	0.357	0.122	0.296	0.106	0.381	0.001	0.751	0.003	0.5	-0.002	0.603	-0.002	0.606	9	129566028	ZBTB43;ZBTB43	N_Shore
cg00979628	-0.028	0.815	0.175	0.143	0.057	0.628	0.114	0.343	0.002	0.16	0.002	0.141	-0.001	0.58	0	0.995	19	1355239	MUM1;MUM1	Island
cg00997172	-0.097	0.409	-0.09	0.454	-0.053	0.653	0.006	0.958	-0.001	0.581	0	0.804	0	0.719	0.001	0.462	20	32399000	CHMP4B	N_Shore
cg01008495	-0.217	0.064	-0.106	0.377	-0.151	0.197	-0.018	0.879	0.004	0.155	0.002	0.439	0.001	0.538	-0.001	0.52	18	43330032	SLC14A1;SLC14A1;SLC14A1;SLC14A1	

cg01013732	-0.139	0.236	0.186	0.121	0.008	0.946	-0.057	0.636	-0.003	0.297	0	0.949	-0.003	0.187	-0.002	0.369	20	44992585	SLC35C2;SLC35C2;SLC35C2	N_Shore
cg01080375	-0.141	0.231	0.071	0.556	-0.109	0.35	0.019	0.876	0	0.93	0	0.796	0.001	0.559	-0.001	0.158	14	71109264	TTC9	Island
cg01122304	-0.052	0.659	-0.009	0.941	-0.052	0.657	0.02	0.866	-0.011	0.19	-0.011	0.201	-0.011	0.172	-0.011	0.204	4	2845576	ADD1;ADD1;ADD1;ADD1	Island
cg01124027	0.027	0.819	0.063	0.602	-0.151	0.197	-0.003	0.981	0	0.966	0.001	0.464	-0.001	0.316	0	0.724	4	4543907	STX18	Island
cg01125333	0.018	0.88	0.068	0.572	-0.048	0.684	0.203	0.09	0	0.914	0.001	0.214	0	0.892	0	0.839	16	2835331	PRSS33	Island
cg01173978	0.031	0.795	-0.159	0.186	-0.043	0.714	-0.038	0.753	-0.001	0.897	-0.009	0.131	-0.008	0.104	-0.008	0.232	16	14097192		
cg01201519	0.065	0.583	-0.158	0.189	0.219	0.059	0.119	0.325	0.004	0.356	-0.004	0.481	0.002	0.528	0.003	0.462	7	121950429		Island
cg01230160	0.008	0.949	0.034	0.781	-0.099	0.398	-0.097	0.421	0.002	0.339	0	0.982	0	0.985	0.003	0.103	2	234263768	DGKD	
cg01273580	0.052	0.662	-0.083	0.492	-0.027	0.821	0.014	0.907	0.007	0.14	0.006	0.192	0.006	0.211	0.001	0.838	19	49946108	SLC17A7	N_Shore
cg01306614	0.094	0.427	-0.053	0.663	-0.198	0.089	0.026	0.827	0	0.936	0.002	0.131	-0.002	0.104	-0.001	0.383	5	72112331	TNPO1	Island
cg01313518	0.032	0.789	-0.013	0.916	0.117	0.316	-0.082	0.498	0.002	0.11	-0.002	0.113	-0.001	0.418	0.001	0.436	2	25584954		
cg01346114	0.068	0.563	-0.013	0.916	-0.089	0.446	-0.137	0.255	-0.001	0.435	0.002	0.112	-0.001	0.475	0	0.979	17	7218875	GPS2	Island
cg01441593	0.088	0.458	0.156	0.193	0.137	0.24	0.14	0.244	-0.001	0.523	0.002	0.197	-0.001	0.397	0	0.947	17	79818808	P4HB	Island
cg01489519	-0.185	0.115	0.028	0.814	0.158	0.175	-0.158	0.188	0.002	0.142	0	0.826	-0.001	0.156	0.002	0.102	20	61493186	TCFL5	Island
cg01495907	0.168	0.153	0.124	0.302	-0.212	0.068	0.053	0.659	0.003	0.217	0.004	0.132	-0.004	0.152	-0.001	0.643	14	24842974	NFATC4;NFATC4	S_Shelf
cg01544807	-0.005	0.965	0.058	0.63	-0.074	0.528	-0.084	0.484	0	0.93	-0.001	0.445	-0.002	0.12	0.001	0.476	8	42195539	POLB	N_Shore
cg01553946	-0.142	0.227	0.055	0.646	0.1	0.395	-0.103	0.393	-0.002	0.508	0.001	0.684	-0.001	0.585	0.001	0.594	5	149828936	RPS14;RPS14;RPS14	Island
cg01588060	-0.032	0.787	0.127	0.291	0.146	0.212	0.018	0.88	-0.003	0.25	-0.001	0.716	-0.004	0.103	-0.002	0.346	6	2245749	GMDS;GMDS	Island
cg01625044	0.149	0.206	0.067	0.578	0.11	0.348	0.048	0.692	-0.003	0.193	0.001	0.661	-0.001	0.774	-0.003	0.187	6	28963898	ZNF311	
cg01669366	-0.024	0.84	-0.014	0.907	-0.118	0.315	0.096	0.425	0.002	0.524	-0.003	0.249	-0.005	0.157	-0.005	0.139	7	100077394	TSC22D4	S_Shore
cg01671059	-0.188	0.109	-0.043	0.722	0.051	0.667	-0.052	0.667	-0.002	0.206	0	0.633	-0.002	0.11	-0.001	0.542	8	73921086	TERF1;TERF1	Island
cg01677987	-0.051	0.665	-0.079	0.511	0.081	0.492	-0.083	0.494	0.002	0.365	-0.003	0.351	-0.002	0.365	-0.005	0.1	16	341404	AXIN1;AXIN1	Island
cg01746873	0.002	0.99	0.082	0.499	0.058	0.621	-0.206	0.085	0.003	0.272	-0.002	0.255	-0.003	0.115	0.003	0.405	13	113344027	ATP11A;ATP11A	Island
cg01809845	-0.006	0.958	0.01	0.932	0.018	0.88	0.09	0.455	-0.002	0.118	0.001	0.5	0.002	0.284	0	0.99	5	153418307	FAM114A2;MFAP3;MFAP3;MFAP3	Island
cg01825986	0.113	0.336	-0.127	0.291	0.061	0.605	0.031	0.796	0	0.618	-0.001	0.39	0	0.698	0.002	0.179	20	47804552	STAU1;STAU1;STAU1;STAU1;STAU1	Island
cg01926858	-0.095	0.421	-0.041	0.732	-0.115	0.324	-0.037	0.761	-0.02	0.12	-0.019	0.154	-0.02	0.105	-0.019	0.156	14	53619558	DDHD1;DDHD1;DDHD1	Island
cg01939414	0.086	0.467	-0.007	0.957	0.198	0.088	0.132	0.271	0.001	0.628	-0.001	0.514	-0.002	0.299	-0.002	0.374	2	103353548	MFSD9	S_Shore
cg01947585	0.056	0.633	-0.065	0.588	0.085	0.468	0.013	0.912	-0.001	0.131	0.001	0.454	-0.001	0.112	0	0.934	15	59225861	SLTM;SLTM	Island
cg01948390	-0.143	0.223	0.004	0.974	0.018	0.877	0.002	0.987	-0.001	0.545	0.002	0.126	-0.002	0.116	0.001	0.647	11	22850866	SVIP	Island

cg01980562	0.142	0.228	0.12	0.317	0.059	0.618	-0.09	0.458	-0.001	0.837	0.004	0.223	-0.004	0.216	-0.002	0.587	19	1174207	SBNO2;SBNO2	Island
cg01985852	0.054	0.647	-0.057	0.639	-0.065	0.577	-0.098	0.416	0.001	0.211	0.001	0.359	-0.002	0.223	0	0.987	16	88772985	CTU2;RNF166;CTU2	Island
cg01988550	0.168	0.153	0.035	0.771	-0.033	0.779	-0.182	0.129	-0.002	0.119	0.001	0.695	-0.002	0.125	-0.001	0.358	7	28966659		
cg02013961	0.132	0.263	-0.074	0.54	-0.144	0.218	0.041	0.736	-0.001	0.488	0.003	0.125	0	0.978	-0.001	0.698	6	142468197	VTA1	Island
cg02032696	-0.157	0.181	0.184	0.125	-0.016	0.892	0	0.998	0	0.61	0	0.867	-0.001	0.402	0	0.814	14	67982198	TMEM229B	Island
cg02058552	-0.214	0.067	-0.039	0.746	0.118	0.315	-0.033	0.788	0	0.753	-0.002	0.176	-0.001	0.401	0	0.943	10	5454564	NET1;NET1	Island
cg02067846	0.073	0.537	0.151	0.21	-0.054	0.645	-0.107	0.376	0	0.987	0.001	0.316	0	0.711	0	0.91	3	38071498	PLCD1	Island
cg02079741	0.129	0.274	0.048	0.689	0.162	0.165	0.076	0.527	0.006	0.428	-0.003	0.681	-0.01	0.22	0.009	0.303	2	25390424	POMC;POMC	N_Shore
cg02090806	-0.039	0.744	0.024	0.843	-0.106	0.367	-0.093	0.441	0.001	0.333	0.002	0.211	-0.001	0.37	0.002	0.226	6	33239496	VPS52;RPS18;VPS52	Island
cg02097588	0.186	0.113	-0.009	0.939	-0.121	0.301	0.064	0.599	0	0.799	0.001	0.237	-0.001	0.314	0.001	0.168	2	10830232	NOL10	Island
cg02145101	0.091	0.442	0.025	0.838	-0.183	0.116	-0.108	0.368	0	0.79	0.001	0.297	-0.001	0.245	0	0.643	14	24768773	DHRS1;C14orf21;DHRS1	Island
cg02154956	0.201	0.086	0.053	0.66	-0.075	0.525	-0.115	0.34	0	0.965	0	0.882	-0.001	0.154	-0.001	0.318	13	100741410	PCCA;PCCA	Island
cg02169333	-0.194	0.097	0.141	0.241	0.052	0.659	0.121	0.313	0.001	0.138	0	0.693	-0.001	0.158	-0.001	0.128	16	57023494	NLRC5;NLRC5	Island
cg02189786	-0.039	0.743	-0.197	0.099	0.102	0.384	0.049	0.684	-0.016	0.161	-0.013	0.272	-0.015	0.187	-0.017	0.15	16	67875959	THAP11;CENPT	Island
cg02392688	0.189	0.107	0.198	0.098	0.108	0.358	0.07	0.562	-0.011	0.327	-0.009	0.439	-0.018	0.184	-0.019	0.191	17	27916351	GIT1;GIT1	Island
cg02472801	0.042	0.724	0.213	0.075	0.142	0.223	-0.043	0.724	0.012	0.433	0.012	0.373	-0.002	0.88	-0.015	0.441	8	2480483		Island
cg02476028	-0.072	0.542	0.172	0.153	0.135	0.247	-0.004	0.971	-0.001	0.708	0.001	0.645	-0.003	0.294	0	0.976	11	17035779	PLEKHA7	Island
cg02496111	-0.159	0.175	-0.045	0.71	-0.103	0.38	0.169	0.158	0.001	0.222	0	0.693	-0.001	0.41	0	0.918	16	3156779		Island
cg02500267	0.181	0.124	0.13	0.279	-0.048	0.686	-0.01	0.937	0	0.932	0.001	0.626	-0.001	0.494	0.001	0.666	20	18568541	DTD1	Island
cg02506043	0.14	0.234	0.116	0.336	0.122	0.298	0.149	0.215	-0.003	0.335	0	0.871	-0.003	0.31	-0.003	0.295	1	17765068	RCC2;RCC2	Island
cg02509647	0.063	0.593	0.141	0.242	-0.009	0.939	0.023	0.852	0.001	0.559	0.002	0.109	0	0.992	-0.001	0.436	8	94712810	FAM92A1;FAM92A1	Island
cg02547025	-0.085	0.47	-0.121	0.314	-0.043	0.714	-0.012	0.921	0.003	0.196	0	0.801	0	0.909	0.001	0.803	2	30454275	LBH	Island
cg02560642	-0.01	0.935	-0.055	0.646	-0.138	0.239	-0.017	0.89	0.001	0.759	-0.003	0.353	-0.001	0.659	0.004	0.16	13	110664301		
cg02598341	-0.09	0.447	-0.082	0.497	0.049	0.674	-0.165	0.17	-0.003	0.156	0.002	0.355	0	0.842	-0.002	0.301	15	31618618	KLF13	Island
cg02661802	0.077	0.514	0.165	0.168	0.071	0.546	-0.151	0.209	0	0.834	0.001	0.534	-0.001	0.137	0	0.656	10	124221056	HTRA1;HTRA1	Island
cg02684904	0.042	0.723	0.209	0.08	-0.026	0.826	0.07	0.563	-0.001	0.725	0	0.991	-0.002	0.503	-0.003	0.239	17	38137393	PSMD3	Island
cg02776035	0.032	0.787	-0.008	0.949	0.027	0.819	0.041	0.735	0.002	0.501	-0.001	0.854	-0.003	0.267	0.005	0.105	19	51161482		N_Shore
cg02803139	-0.054	0.646	0.068	0.575	-0.105	0.369	-0.041	0.734	0.002	0.514	0.001	0.64	-0.003	0.177	0.004	0.133	10	106113391	CCDC147	
cg02842381	-0.09	0.447	-0.014	0.907	0.108	0.354	0.133	0.268	0.001	0.421	0.001	0.175	0	0.957	0	0.713	8	145634766	CPSF1	Island
cg02853781	0.055	0.641	0.07	0.562	0.202	0.083	0.088	0.466	0.001	0.447	0.001	0.169	0	0.675	0.001	0.105	1	19229053	ALDH4A1;ALDH4A1;ALDH4A1;ALDH4A1	Island

cg02858053	0.004	0.976	0.049	0.683	-0.017	0.887	0.013	0.916	-0.001	0.157	0.001	0.131	-0.001	0.514	0	0.701	1	84971927	SPATA1;GNG5	Island
cg02862904	0.222	0.058	0.031	0.798	-0.043	0.713	0.092	0.444	-0.002	0.122	0	0.89	0	0.79	0.001	0.359	16	5540758		
