# Supplementary material for: Interindividual methylomic variation across blood, cortex, and cerebellum: implications for epigenetic studies of neurological and neuropsychiatric phenotypes
Source: Epigenetics. 2015 Oct 12;10(11):1024–32. doi: 10.1080/15592294.2015.1100786 (PMC4844197; doi:10.1080/15592294.2015.1100786)
Supplement: 1100786_Supplemental_Material.zip [file kepi-10-11-1100786-s001.zip › Table S7.pdf]

ProbeID	Blood PFC correlation		Blood EC correlation		Blood STG correlation		Blood CER correlation		Difference between blood and PFC		Difference between blood and EC		Difference between blood and STG		Difference between blood and CER		CHR	MAPINFO	UCSC_REFGENE_NAME	RELATION_TO_UCSC_CPG_ISLAND
	r	p value	r	p value	r	p value	r	p value	Mean diff.	P value	Mean diff.	P value	Mean diff.	P value	Mean diff.	P value				
cg00008695	0.814	0	0.852	0	0.836	0	0.879	0	0.059	0	0.064	0	0.054	0	0.087	0	8	960721		N_Shore
cg00008932	0.895	0	0.865	0	0.887	0	0.887	0	0.064	0	0.06	0	0.05	0	0.079	0	23	71498597	RPS4X	S_Shore
cg00012317	0.902	0	0.928	0	0.942	0	0.9	0	0.083	0	0.08	0	0.078	0	0.133	0	1	149370974	FCGR1C	
cg00013655	0.877	0	0.852	0	0.887	0	0.931	0	0.2	0	0.195	0	0.185	0	0.107	0	16	629015	PIGQ;PIGQ	N_Shore
cg00016522	0.899	0	0.891	0	0.922	0	0.932	0	0.173	0	0.131	0	0.164	0	0.119	0	23	149737876	MTM1	S_Shore
cg00021325	0.909	0	0.92	0	0.91	0	0.905	0	-0.099	0	-0.087	0	-0.099	0	-0.077	0	14	91294412		
cg00040455	0.937	0	0.921	0	0.928	0	0.917	0	-0.055	0	-0.053	0	-0.06	0	-0.078	0	23	30326676	NR0B1	Island
cg00053135	0.962	0	0.968	0	0.962	0	0.964	0	0.034	0	0.026	0	0.026	0	0.035	0	6	167032403	RPS6KA2;RPS6KA2	Island
cg00114966	0.869	0	0.864	0	0.887	0	0.89	0	0.176	0	0.161	0	0.163	0	0.206	0	1	197893920	LHX9;LHX9	S_Shelf
cg00123128	0.759	0	0.75	0	0.813	0	-0.725	0	-0.09	0	-0.091	0	-0.078	0	-0.472	0	4	3464702	DOK7;DOK7	N_Shore
cg00130710	0.856	0	0.844	0	0.816	0	0.855	0	0.061	0	0.061	0	0.056	0	0.087	0	6	40565925		N_Shore
cg00159953	0.974	0	0.97	0	0.954	0	0.962	0	-0.015	0	-0.019	0	-0.023	0	-0.037	0	21	47547796	COL6A2;COL6A2;COL6A2	N_Shore
cg00188089	0.946	0	0.935	0	0.935	0	0.971	0	-0.071	0	-0.045	0	-0.085	0	0.024	0	6	135027966		
cg00192980	0.991	0	0.991	0	0.99	0	0.988	0	-0.017	0	-0.025	0	-0.022	0	-0.039	0	23	77394971	TAF9B	Island
cg00197266	0.86	0	0.883	0	0.855	0	0.818	0	0.083	0	0.086	0	0.065	0	0.101	0	6	22567723		N_Shore
cg00238131	0.873	0	0.825	0	0.893	0	0.794	0	-0.042	0	-0.035	0	-0.045	0	-0.09	0	23	71527298	CITED1;CITED1	Island
cg00242950	0.972	0	0.975	0	0.978	0	0.98	0	0.025	0	0.019	0	0.018	0	0.026	0	3	112899162		
cg00253811	0.935	0	0.935	0	0.949	0	0.949	0	0.043	0	0.039	0	0.032	0	0.041	0	23	15353923	PIGA;PIGA	Island
cg00257187	0.921	0	0.904	0	0.897	0	0.904	0	0.03	0	0.026	0	0.023	0	0.03	0	10	91401349	PANK1;PANK1;PANK1	N_Shelf
cg00277334	0.926	0	0.889	0	0.895	0	0.784	0	-0.031	0	-0.022	0	-0.041	0	0.221	0	10	82204260		
cg00286512	0.761	0	0.892	0	0.81	0	0.821	0	0.053	0	0.043	0	0.043	0	0.043	0	7	754102	PRKAR1B;PRKAR1B;PRKAR1B;PRKAR1B	S_Shore
cg00290607	0.79	0	0.756	0	0.772	0	0.762	0	-0.051	0	-0.082	0	-0.07	0	-0.301	0	11	67383545		Island
cg00314943	0.931	0	0.907	0	0.894	0	0.906	0	0.043	0	0.029	0	0.029	0	0.042	0	2	233270750	ALPPL2	N_Shore
cg00316478	0.886	0	0.736	0	0.886	0	0.917	0	-0.156	0	-0.161	0	-0.164	0	-0.086	0	16	89222027	ACSF3	N_Shelf
cg00322666	0.767	0	0.749	0	0.74	0	0.717	0	0.023	0	0.026	0	0.02	0	0.032	0	3	87994995		
cg00337921	0.981	0	0.977	0	0.979	0	0.972	0	0.044	0	0.038	0	0.03	0	0.041	0	23	129091288		Island
cg00356251	0.864	0	0.889	0	0.885	0	0.92	0	0.029	0	0.019	0	0.022	0	0.052	0	7	158051019	PTPRN2;PTPRN2;PTPRN2	
cg00356916	0.816	0	0.81	0	0.747	0	0.748	0	0.078	0	0.072	0	0.064	0	0.045	0	1	116256618	CASQ2	
cg00374346	0.753	0	0.785	0	0.798	0	-0.78	0	0.082	0	0.066	0	0.06	0	-0.336	0	23	71349037	NHSL2;RGAG4;RGAG4	N_Shore

cg00375457	0.76	0	0.735	0	0.739	0	0.759	0	0.141	0	0.132	0	0.133	0	0.117	0	11	5617273	TRIM6-TRIM34;TRIM6;TRIM6	
cg00379371	0.752	0	0.717	0	0.749	0	0.719	0	0.092	0	0.095	0	0.086	0	0.073	0	23	48774742	PIM2	N_Shore
cg00389552	0.889	0	0.897	0	0.896	0	0.889	0	0.126	0	0.12	0	0.113	0	0.154	0	23	40028487	BCOR;BCOR	N_Shore
cg00403716	0.755	0	0.754	0	0.779	0	0.791	0	0.196	0	0.195	0	0.192	0	0.176	0	6	167789441	TCP10	
cg00408231	0.764	0	0.767	0	0.773	0	0.773	0	-0.11	0	-0.101	0	-0.113	0	-0.112	0	23	100807848	ARMCX1	Island
cg00412010	0.939	0	0.935	0	0.927	0	0.907	0	-0.069	0	-0.039	0	-0.054	0	-0.097	0	23	110339837	PAK3;PAK3;PAK3	S_Shore
cg00423704	0.77	0	0.765	0	0.714	0	0.766	0	0.068	0	0.067	0	0.062	0	0.065	0	20	21077665		N_Shelf
cg00426668	0.913	0	0.897	0	0.915	0	0.905	0	0.099	0	0.102	0	0.092	0	0.147	0	23	110514231	CAPN6	
cg00430613	0.861	0	0.857	0	0.841	0	0.812	0	0.091	0	0.071	0	0.087	0	0.096	0	17	406426		Island
cg00432233	0.832	0	0.739	0	0.748	0	0.747	0	0.032	0	0.026	0	0.022	0	0.033	0	6	6292977	F13A1	
cg00432461	0.781	0	0.777	0	0.79	0	0.799	0	-0.039	0	-0.046	0	-0.048	0	-0.028	0	8	37825156	ADRB3	S_Shore
cg00438309	0.949	0	0.944	0	0.956	0	0.945	0	0.023	0	0.032	0	0.021	0	0.061	0	1	36620222		N_Shore
cg00447581	0.838	0	0.836	0	0.839	0	0.847	0	0.028	0	0.015	0	0.012	0	0.025	0	3	52724578	GNL3;GNL3;GNL3;SNORD19B	
cg00453258	0.736	0	0.749	0	0.737	0	0.765	0	0.069	0	0.093	0	0.059	0	0.088	0	10	105219172	CALHM1	S_Shore
cg00484940	0.94	0	0.933	0	0.946	0	0.926	0	-0.039	0	-0.034	0	-0.05	0	-0.053	0	23	85403143	DACH2;DACH2	Island
cg00490603	0.902	0	0.87	0	0.929	0	0.921	0	-0.113	0	-0.162	0	-0.111	0	-0.027	0	22	46262041		N_Shore
cg00507855	0.913	0	0.933	0	0.938	0	0.948	0	0.047	0	0.051	0	0.04	0	0.052	0	20	45605303	EYA2;EYA2	
cg00512484	0.768	0	0.76	0	0.74	0	0.734	0	0.11	0	0.115	0	0.096	0	0.159	0	18	5199989		S_Shelf
cg00518468	0.941	0	0.94	0	0.956	0	0.892	0	0.031	0	0.019	0	0.021	0	0.018	0	9	139588064		N_Shore
cg00525383	0.91	0	0.911	0	0.921	0	0.887	0	-0.053	0	-0.034	0	-0.056	0	-0.103	0	23	136647793	ZIC3	Island
cg00554969	0.964	0	0.967	0	0.963	0	0.957	0	-0.038	0	-0.033	0	-0.035	0	-0.063	0	23	79278012	TBX22;TBX22;TBX22	
cg00567190	0.821	0	0.791	0	0.821	0	0.729	0	-0.128	0	-0.123	0	-0.137	0	-0.142	0	1	211556508	C1orf97	S_Shore
cg00584971	0.881	0	0.862	0	0.867	0	0.858	0	-0.065	0	-0.061	0	-0.071	0	-0.066	0	23	99665588	PCDH19;PCDH19	N_Shore
cg00622389	0.956	0	0.936	0	0.956	0	0.947	0	-0.055	0	-0.052	0	-0.06	0	-0.094	0	23	100914950	ARMCX2;ARMCX2	
cg00625841	0.893	0	0.855	0	0.882	0	0.805	0	-0.05	0	-0.039	0	-0.051	0	-0.061	0	23	134156624	FAM127C	S_Shore
cg00630870	0.887	0	0.928	0	0.907	0	0.912	0	0.052	0	0.041	0	0.039	0	0.056	0	10	89113843		N_Shore
cg00632374	0.969	0	0.974	0	0.984	0	0.983	0	0.085	0	0.08	0	0.076	0	0.086	0	23	46432929	CHST7	N_Shore
cg00659559	0.756	0	0.741	0	0.797	0	0.791	0	0.043	0	0.081	0	0.045	0	0.216	0	7	29961120	SCRN1;SCRN1;SCRN1;SCRN1	
cg00660096	0.849	0	0.796	0	0.872	0	0.855	0	0.039	0	0.029	0	0.035	0	0.047	0	17	81023323		N_Shore
cg00665405	0.725	0	0.727	0	0.756	0	0.783	0	-0.1	0	-0.094	0	-0.105	0	-0.103	0	12	50899548	DIP2B	S_Shore
cg00688236	0.825	0	0.865	0	0.841	0	0.84	0	0.064	0	0.088	0	0.055	0	0.076	0	8	1921740	KBTBD11	Island
cg00691081	0.82	0	0.776	0	0.788	0	0.833	0	0.051	0	0.054	0	0.045	0	0.062	0	16	6379449	A2BP1;A2BP1	

cg00697486	0.709	0	0.839	0	0.741	0	0.87	0	-0.087	0	-0.038	0	-0.098	0	-0.059	0	1	233224593	PCNXL2	
cg00723973	0.982	0	0.986	0	0.985	0	0.979	0	0.046	0	0.06	0	0.041	0	0.057	0	23	46696265	RP2	Island
cg00727334	0.933	0	0.918	0	0.909	0	0.893	0	0.039	0	0.03	0	0.025	0	-0.018	0	4	57456686		N_Shore
cg00733150	0.91	0	0.924	0	0.877	0	0.897	0	-0.044	0	-0.042	0	-0.04	0	-0.067	0	22	45705707	FAM118A;FAM118A	Island
cg00753039	0.948	0	0.96	0	0.961	0	0.948	0	0.031	0	0.029	0	0.017	0	0.058	0	10	134969141		N_Shelf
cg00753924	0.861	0	0.886	0	0.859	0	0.788	0	-0.169	0	-0.146	0	-0.173	0	-0.053	0	9	137298813	RXRA	N_Shore
cg00770693	0.76	0	0.75	0	0.793	0	0.768	0	0.025	0	0.025	0	0.027	0	0.064	0	12	132932098		S_Shore
cg00779294	0.882	0	0.785	0	0.746	0	0.887	0	-0.02	0	-0.069	0	-0.064	0	-0.021	0	3	87325752	POU1F1;POU1F1	
cg00787015	0.856	0	0.838	0	0.848	0	0.828	0	0.048	0	0.057	0	0.046	0	0.033	0	23	40506177	CXorf38	Island
cg00809502	0.885	0	0.858	0	0.844	0	0.893	0	0.029	0	0.022	0	0.015	0	0.024	0	13	106142193	DAOA;DAOA;DAOA	
cg00815399	0.791	0	0.812	0	0.797	0	0.73	0	0.146	0	0.11	0	0.108	0	-0.061	0	7	158750607		N_Shore
cg00829575	0.961	0	0.966	0	0.962	0	0.96	0	0.056	0	0.055	0	0.039	0	0.07	0	23	38663062	MID1IP1;MID1IP1;MID1IP1	Island
cg00832270	0.991	0	0.994	0	0.994	0	0.991	0	0.021	0	0.022	0	0.021	0	0.018	0	23	77359535	PGK1	Island
cg00837987	0.969	0	0.974	0	0.963	0	0.968	0	0.047	0	0.039	0	0.046	0	0.039	0	8	588849		
cg00863716	0.887	0	0.898	0	0.856	0	0.87	0	0.088	0	0.113	0	0.1	0	0.078	0	10	5061094	AKR1C2;AKR1C2	
cg00867835	0.869	0	0.887	0	0.893	0	0.866	0	0.08	0	0.058	0	0.069	0	0.048	0	7	149484985	SSPO	N_Shelf
cg00879592	0.884	0	0.877	0	0.834	0	0.819	0	0.032	0	0.035	0	0.023	0	0.036	0	17	62400642	PECAM1;PECAM1	
cg00885682	0.886	0	0.892	0	0.889	0	0.907	0	-0.054	0	-0.044	0	-0.055	0	-0.062	0	16	22825749	HS3ST2	Island
cg00914147	0.891	0	0.882	0	0.876	0	0.835	0	0.02	0	0.024	0	0.032	0	0.052	0	17	973405	ABR;ABR;ABR	N_Shelf
cg00917018	0.964	0	0.965	0	0.969	0	0.97	0	0.043	0	0.042	0	0.033	0	0.05	0	23	140271549	LDOC1	Island
cg00951395	0.782	0	0.798	0	0.758	0	0.739	0	-0.143	0	-0.104	0	-0.133	0	-0.241	0	1	232941775	KIAA1383	S_Shore
cg00953403	0.817	0	0.816	0	0.78	0	0.79	0	-0.011	0	-0.011	0	-0.012	0	-0.022	0	17	74099816	EXOC7;EXOC7;EXOC7;EXOC7;EXOC7;EXOC7;EXOC7;EXOC7;EXOC7;EXOC7;EXOC7;EXOC7;EXOC7	Island
cg00960898	0.911	0	0.953	0	0.901	0	0.901	0	-0.042	0	-0.041	0	-0.054	0	-0.06	0	23	136113605	GPR101	Island
cg00972140	0.777	0	0.826	0	0.822	0	0.832	0	0.03	0	0.027	0	0.021	0	0.033	0	1	5037068		
cg00973938	0.908	0	0.889	0	0.871	0	0.856	0	0.025	0	0.03	0	0.022	0	0.021	0	16	89654405	CPNE7;CPNE7	Island
cg01026744	0.978	0	0.974	0	0.979	0	0.976	0	0.026	0	0.02	0	0.015	0	0.032	0	4	89619053	NAP1L5;HERC3	
cg01039990	0.933	0	0.979	0	0.984	0	0.97	0	0.102	0	0.037	0	0.032	0	0.051	0	23	136510831		Island

cg01049849	0.711	0	0.714	0	0.784	0	0.795	0	0.142	0	0.137	0	0.143	0	0.173	0	1	10807676	CASZ1;CASZ1	
cg01050423	0.842	0	0.799	0	0.805	0	0.865	0	-0.027	0	-0.034	0	-0.033	0	-0.03	0	2	103236078	SLC9A2	Island
cg01056373	0.978	0	0.98	0	0.98	0	0.978	0	0.041	0	0.039	0	0.039	0	0.041	0	23	151999547	NSDHL;NSDHL;NSDHL;NSDHL;CETN2	Island
cg01058932	0.751	0	0.769	0	0.738	0	0.739	0	-0.062	0	-0.067	0	-0.07	0	-0.078	0	23	55021626	PFKFB1	
cg01062068	0.862	0	0.873	0	0.865	0	0.844	0	-0.036	0	-0.025	0	-0.041	0	-0.049	0	16	2039821	SYNGR3	N_Shore
cg01070560	0.96	0	0.932	0	0.958	0	0.961	0	0.043	0	0.037	0	0.03	0	0.039	0	8	3047756	CSMD1	
cg01079515	0.944	0	0.937	0	0.939	0	0.877	0	-0.08	0	-0.067	0	-0.065	0	-0.11	0	3	195576629		
cg01082559	0.878	0	0.854	0	0.894	0	0.872	0	0.057	0	0.048	0	0.047	0	0.064	0	20	49622513	KCNG1	S_Shore
cg01085553	0.812	0	0.9	0	0.944	0	0.968	0	-0.049	0	-0.045	0	-0.044	0	0.014	0	15	81225491	KIAA1199	
cg01116831	0.835	0	0.838	0	0.859	0	0.832	0	0.046	0	0.042	0	0.037	0	0.056	0	16	2190356		S_Shelf
cg01131100	0.851	0	0.83	0	0.88	0	0.906	0	0.058	0	0.055	0	0.047	0	0.082	0	14	101495047	MIR494	
cg01164094	0.893	0	0.88	0	0.918	0	0.924	0	0.031	0	0.021	0	0.023	0	0.053	0	12	104230671	NT5DC3	N_Shelf
