# Supplementary material for: Interindividual methylomic variation across blood, cortex, and cerebellum: implications for epigenetic studies of neurological and neuropsychiatric phenotypes
Source: Epigenetics. 2015 Oct 12;10(11):1024–32. doi: 10.1080/15592294.2015.1100786 (PMC4844197; doi:10.1080/15592294.2015.1100786)
Supplement: 1100786_Supplemental_Material.zip [file kepi-10-11-1100786-s001.zip › Table S8.pdf]

ProbeID	CHR	MAPINFO	UCSC_REFGENE_NAME	UCSC_REFGENE_GROUP	RELATION_TO_UCSC_CPG_ISLAND	Blood stable	PFC variable	EC variable	STG variable	CER variable
cg13864868	9	95820799	SUSD3	TSS200	Island	TRUE	TRUE	TRUE	TRUE	TRUE
cg04916091	19	17958647	JAK3	5'UTR	Island	TRUE	TRUE	TRUE	TRUE	TRUE
cg22197033	17	8649004	CCDC42;CCDC42	TSS1500;TSS1500	Island	TRUE	TRUE	TRUE	TRUE	FALSE
cg02567182	8	94746420	RBM12B	Body		TRUE	TRUE	TRUE	TRUE	FALSE
cg17251196	6	33288599	DAXX;DAXX;DAXX;DAXX	Body;Body;Body;Body	N_Shore	TRUE	TRUE	TRUE	FALSE	TRUE
cg18676237	6	2903258	SERPINB9	5'UTR	Island	TRUE	FALSE	TRUE	FALSE	FALSE
cg22713976	10	50815015			N_Shelf	TRUE	FALSE	FALSE	FALSE	TRUE
cg16107483	1	28199456	C1orf38;C1orf38;C1orf38	Body;Body;Body	S_Shore	TRUE	TRUE	FALSE	FALSE	FALSE
cg26967566	16	85743863	C16orf74	Body	Island	TRUE	FALSE	TRUE	FALSE	FALSE
cg09887589	6	31702785	CLIC1	Body		TRUE	TRUE	TRUE	TRUE	TRUE
cg03548062	14	101158251				TRUE	TRUE	TRUE	TRUE	TRUE
cg24983367	17	58228482	CA4	Body	S_Shore	TRUE	TRUE	TRUE	TRUE	FALSE
cg19463665	13	113492776	ATP11A;ATP11A	Body;Body	Island	TRUE	FALSE	FALSE	FALSE	TRUE
cg12786570	10	30316432	KIAA1462	Body		TRUE	TRUE	TRUE	TRUE	TRUE
cg07560305	1	89461312			S_Shelf	TRUE	TRUE	TRUE	TRUE	TRUE
cg12940747	11	128595713	FLI1;FLI1	Body;5'UTR		TRUE	TRUE	TRUE	TRUE	TRUE
cg20936604	3	58311152				TRUE	TRUE	TRUE	FALSE	TRUE
cg12315995	15	25920224				TRUE	TRUE	TRUE	TRUE	TRUE
cg26148377	1	153605715	C1orf77;S100A13	TSS1500;5'UTR	N_Shore	TRUE	TRUE	TRUE	TRUE	TRUE
cg21415347	1	22777839	ZBTB40;ZBTB40	TSS1500;TSS1500	N_Shore	TRUE	TRUE	TRUE	TRUE	FALSE
cg09778867	12	53718166			N_Shore	TRUE	TRUE	TRUE	TRUE	TRUE
cg02395479	3	53106725				TRUE	TRUE	TRUE	TRUE	TRUE
cg13055001	11	67169813	PPP1CA;PPP1CA;PPP1CA	TSS1500;TSS1500;TSS1500	S_Shore	TRUE	TRUE	TRUE	TRUE	TRUE
cg07097863	1	202162150	LGR6	TSS1500	N_Shore	TRUE	TRUE	TRUE	TRUE	TRUE
cg15946904	17	957248	ABR;ABR;ABR	Body;Body;Body		TRUE	TRUE	TRUE	TRUE	FALSE
cg00537979	21	46876731	COL18A1;COL18A1;COL18A1	Body;Body;1stExon	Island	TRUE	TRUE	TRUE	TRUE	FALSE
cg01676844	16	1828146	SPSB3	Body	S_Shore	TRUE	TRUE	TRUE	TRUE	TRUE
cg04230403	10	90750176	FAS;FAS;FAS;FAS;FAS;FAS;FAS;ACTA2	TSS200;TSS200;TSS200;TSS200;TSS200;TSS200;TSS200;5'UTR	N_Shore	TRUE	TRUE	TRUE	TRUE	TRUE
cg17858500	20	3734725	C20orf27	Body	S_Shore	TRUE	TRUE	TRUE	TRUE	FALSE
cg17557540	6	31166174	HCG27	Body	S_Shore	TRUE	TRUE	TRUE	TRUE	TRUE
cg03560685	18	74729551	MBP;MBP;MBP;MBP;MBP;MBP	TSS1500;TSS1500;Body;Body;TSS1500;TSS1500		TRUE	TRUE	TRUE	TRUE	FALSE
cg02454464	19	49957206	ALDH16A1;ALDH16A1	Body;Body	S_Shore	TRUE	TRUE	TRUE	TRUE	TRUE

cg27290274	1	15386739	KIAA1026;KIAA1026;KIAA1026;KIAA1026;KIAA1026	Body;Body;Body;Body;Body		TRUE	TRUE	FALSE	FALSE	FALSE
cg04586407	16	31008574	STX1B	Body	N_Shore	TRUE	TRUE	TRUE	TRUE	FALSE
cg04362907	1	211688645			Island	TRUE	TRUE	TRUE	TRUE	TRUE
cg24578679	15	74658573	CYP11A1;CYP11A1	Body;TSS200	Island	TRUE	FALSE	TRUE	TRUE	TRUE
cg02507181	12	10251536	CLEC1A;CLEC1A	5'UTR;1stExon		TRUE	TRUE	TRUE	TRUE	TRUE
cg25780982	11	8752389	ST5;ST5;ST5	Body;Body;Body	Island	TRUE	TRUE	TRUE	TRUE	FALSE
cg09750385	4	156588012	GUCY1A3;GUCY1A3;GUCY1A3;GUCY1A3;GUCY1A3;GUCY1A3;GUCY1A3;GUCY1A3;GUCY1A3;GUCY1A3;GUCY1A3	1stExon;5'UTR;TSS200;5'UTR;5'UTR;1stExon;5'UTR;TSS200;TSS1500;1stExon;1stExon	N_Shore	TRUE	TRUE	TRUE	FALSE	FALSE
cg18244874	11	130013388	APLP2;APLP2;APLP2;APLP2;APLP2;APLP2	Body;Body;3'UTR;3'UTR;3'UTR;3'UTR		TRUE	FALSE	FALSE	FALSE	TRUE
cg21220614	22	43266108			Island	TRUE	TRUE	TRUE	TRUE	TRUE
cg18038269	19	10464320	TYK2	Body	Island	TRUE	TRUE	TRUE	TRUE	FALSE
cg20441502	19	35633794	FXYD7;FXYD1;FXYD1	TSS1500;3'UTR;3'UTR	N_Shore	TRUE	TRUE	TRUE	TRUE	FALSE
cg06097659	1	1141991	TNFRSF18;TNFRSF18;TNFRSF18;TNFRSF18;TNFRSF18;TNFRSF18	5'UTR;1stExon;5'UTR;1stExon;1stExon;5'UTR	Island	TRUE	TRUE	TRUE	TRUE	TRUE
cg23481748	14	58862503	TOMM20L	TSS200	N_Shore	TRUE	TRUE	TRUE	TRUE	FALSE
cg09154826	1	200635573	DDX59	Body	N_Shelf	TRUE	TRUE	TRUE	TRUE	TRUE
cg16754364	16	29677969	SPN;SPN	3'UTR;3'UTR	S_Shore	TRUE	TRUE	TRUE	TRUE	TRUE
cg07395941	8	1078200				TRUE	TRUE	TRUE	TRUE	FALSE
cg13521538	9	38392470	ALDH1B1	TSS1500	N_Shore	TRUE	TRUE	TRUE	TRUE	TRUE
cg13623638	18	42643746	SETBP1	3'UTR	Island	TRUE	TRUE	TRUE	TRUE	FALSE
cg21197905	11	111602136	PPP2R1B	Body		TRUE	TRUE	TRUE	TRUE	TRUE
cg04999309	2	237018300	AGAP1;AGAP1	Body;Body		TRUE	TRUE	TRUE	TRUE	FALSE
cg11753771	1	214638070	PTPN14	Body		TRUE	TRUE	FALSE	TRUE	FALSE
cg10773698	1	61519473			Island	TRUE	TRUE	TRUE	TRUE	FALSE
cg00664222	8	124934735	FER1L6	5'UTR		TRUE	TRUE	TRUE	TRUE	TRUE
cg27448595	11	875569	CHID1;CHID1;CHID1;CHID1;CHID1	Body;Body;Body;Body;Body		TRUE	FALSE	FALSE	FALSE	TRUE
cg18118033	1	26686703			Island	TRUE	FALSE	FALSE	FALSE	TRUE
cg27376287	21	19192138	C21orf91;C21orf91;C21orf91	TSS1500;TSS1500;TSS1500	S_Shore	TRUE	TRUE	TRUE	FALSE	FALSE
cg07992044	11	68096049	LRP5	Body		TRUE	TRUE	TRUE	TRUE	TRUE
cg20923444	14	105247170	AKT1;AKT1;AKT1	Body;Body;Body		TRUE	TRUE	TRUE	TRUE	TRUE
cg27039467	16	1730561	HN1L	Body	S_Shore	TRUE	TRUE	TRUE	TRUE	TRUE
cg05744675	16	88717723	CYBA	TSS1500	S_Shore	TRUE	TRUE	TRUE	TRUE	TRUE
cg24002183	19	15568574	RASAL3	Body	Island	TRUE	TRUE	TRUE	TRUE	TRUE
cg08077155	11	62435169	C11orf48	Body	S_Shore	TRUE	TRUE	TRUE	TRUE	TRUE

cg14275842	17	4802813	CHRNE;C17orf107	Body;TSS200	Island	TRUE	TRUE	TRUE	TRUE	TRUE
cg14337655	19	2282207	C19orf35	TSS200	S_Shelf	TRUE	TRUE	TRUE	TRUE	TRUE
cg06492111	16	87874392	SLC7A5	Body		TRUE	TRUE	TRUE	TRUE	FALSE
cg18415485	19	3546364	C19orf28;C19orf28;C19orf28	Body;Body;Body	Island	TRUE	FALSE	FALSE	FALSE	TRUE
cg00500522	12	57504387	STAT6	5'UTR		TRUE	TRUE	TRUE	TRUE	TRUE
cg03867475	21	34444382	OLIG1;OLIG1	1stExon;3'UTR	Island	TRUE	FALSE	FALSE	FALSE	TRUE
cg02686681	2	241853954			N_Shore	TRUE	TRUE	TRUE	TRUE	TRUE
cg05688136	2	228882340	SPHKAP;SPHKAP	Body;Body		TRUE	FALSE	FALSE	FALSE	TRUE
cg22907174	20	55201730			Island	TRUE	FALSE	TRUE	FALSE	FALSE
cg21823502	17	79856430	ANAPC11;ANAPC11;ANAPC11;ANAPC11;ANAPC11;ANAPC11;ANAPC11	Body;Body;Body;Body;Body;Body;Body	N_Shelf	TRUE	TRUE	TRUE	TRUE	TRUE
cg14591293	9	130007437			Island	TRUE	FALSE	TRUE	FALSE	FALSE
ch.8.58671533F	8	58508979				TRUE	TRUE	TRUE	TRUE	TRUE
cg26345619	12	2602405	CACNA1C;CACNA1C	Body;Body;Body;Body;Body;Body;Body;Body;Body;Body;Body;Body;Body;Body;Body;Body;Body;Body;Body;Body;Body;Body;Body		TRUE	FALSE	FALSE	FALSE	TRUE
cg09946623	16	29673933	SPN;SPN	TSS1500;TSS1500	N_Shore	TRUE	TRUE	TRUE	TRUE	FALSE
cg00815117	17	27085788	C17orf63;C17orf63	Body;Body		TRUE	FALSE	FALSE	FALSE	TRUE
cg17602167	6	36561558	SFRS3	TSS1500	N_Shore	TRUE	TRUE	TRUE	FALSE	FALSE
cg26033513	3	195595933	TNK2;TNK2	Body;Body	Island	TRUE	FALSE	TRUE	FALSE	FALSE
cg18100007	17	29815661	RAB11FIP4	Body	Island	TRUE	TRUE	TRUE	FALSE	FALSE
cg14482741	17	4802906	CHRNE;C17orf107	Body;TSS200	Island	TRUE	TRUE	TRUE	TRUE	TRUE
cg20943095	15	75123394	CPLX3	3'UTR		TRUE	TRUE	TRUE	TRUE	TRUE
cg13367234	1	17253197	CROCC	Body		TRUE	TRUE	TRUE	TRUE	FALSE
cg17315454	7	75443174	CCL24	TSS200		TRUE	TRUE	TRUE	TRUE	TRUE
cg24594604	1	116947754	C1orf203;C1orf203;C1orf203;C1orf203	Body;Body;Body;Body		TRUE	TRUE	TRUE	TRUE	TRUE
cg02120739	6	89855505	PM20D2	TSS1500	N_Shore	TRUE	FALSE	TRUE	FALSE	FALSE
cg09493150	5	172199605	DUSP1	TSS1500	Island	TRUE	TRUE	TRUE	TRUE	TRUE
cg19212391	17	72432790	GPRC5C;GPRC5C	Body;Body	S_Shelf	TRUE	FALSE	FALSE	FALSE	TRUE
cg22194948	2	43447139				TRUE	TRUE	TRUE	TRUE	TRUE
cg09977852	13	101178575	PCCA;PCCA	Body;Body		TRUE	TRUE	TRUE	TRUE	TRUE
cg16047223	3	195610084	TNK2;TNK2	Body;Body	N_Shelf	TRUE	TRUE	TRUE	TRUE	FALSE
cg10285845	10	74079359			Island	TRUE	TRUE	TRUE	TRUE	TRUE

cg17181598	1	118165662	FAM46C	Body		TRUE	FALSE	FALSE	FALSE	TRUE
cg18328206	1	206681378	RASSF5;RASSF5	1stExon;1stExon	Island	TRUE	TRUE	FALSE	FALSE	FALSE
cg05471373	11	108157868	ATM;ATM	Body;TSS1500		TRUE	TRUE	TRUE	TRUE	FALSE
cg26306372	10	17270232	VIM	TSS200	N_Shore	TRUE	FALSE	FALSE	FALSE	TRUE
cg08814531	6	33280571	TAPBP;TAPBP;TAPBP	Body;Body;Body	N_Shore	TRUE	FALSE	TRUE	FALSE	FALSE
cg17006413	1	942451				TRUE	TRUE	TRUE	TRUE	TRUE
